# Solution plasma synthesis of birnessite-type MnO_2_/carbon composites: support-dependent surface chemistry and alkaline oxygen reduction performance

**DOI:** 10.1039/d6ra06750f

**Published:** 2026-11-02

**Authors:** Kouki Yamamoto, Kentaro Ogihara, Taiyo Suzuki, Toshiki Iwato, Gasidit Panomsuwan, Takahiro Ishizaki

**Affiliations:** a Materials Science and Engineering, Graduate School of Engineering and Science, Shibaura Institute of Technology Toyosu, Koto-Ku Tokyo 135-8548 Japan; b Department of Materials Engineering, Faculty of Engineering, Kasetsart University Bangkok 10900 Thailand; c College of Engineering, Shibaura Institute of Technology Toyosu, Koto-Ku Tokyo 135-8548 Japan ishizaki@shibaura-it.ac.jp

## Abstract

Here, CNT-supported and Ketjen Black (KB)-supported composites were synthesized by a solution plasma process (SPP) for 10 min without external heating. XRD and SEM indicate the formation of poorly crystalline birnessite-type oxide on both supports, with support- and precursor-dependent deposition characteristics. The supports also differ in surface area, surface chemistry, and pretreatment; consequently, this comparison does not isolate morphology as an independent variable. XPS indicates differences in the Mn spectral response, whereas the direction of interfacial charge transfer and the identity of a low-binding-energy C 1s contribution remain unresolved. In O_2_-saturated 0.1 M KOH, KB-M1 exhibited the highest onset potential among the synthesized composites, 0.873 V *vs.* RHE, and a geometric current density of −4.21 mA cm^−2^ at 0.464 V. CNT-M0.5 showed the highest onset potential within the CNT series, 0.804 V. CNT-M0.5 and KB-M1 retained 93.1% and 90.8% of their initial currents, respectively, after 20 000 s of chronoamperometry. The results demonstrate a short SPP route and support-dependent alkaline ORR behavior, while highlighting the need for matched controls and interface-specific measurements to resolve the underlying mechanisms.

## Abstract 2

A 10 min solution plasma treatment produces birnessite-type MnO_2_/carbon composites whose oxide deposition, surface chemistry, and alkaline ORR response vary with the carbon support and precursor concentration.

## Graphical abstract

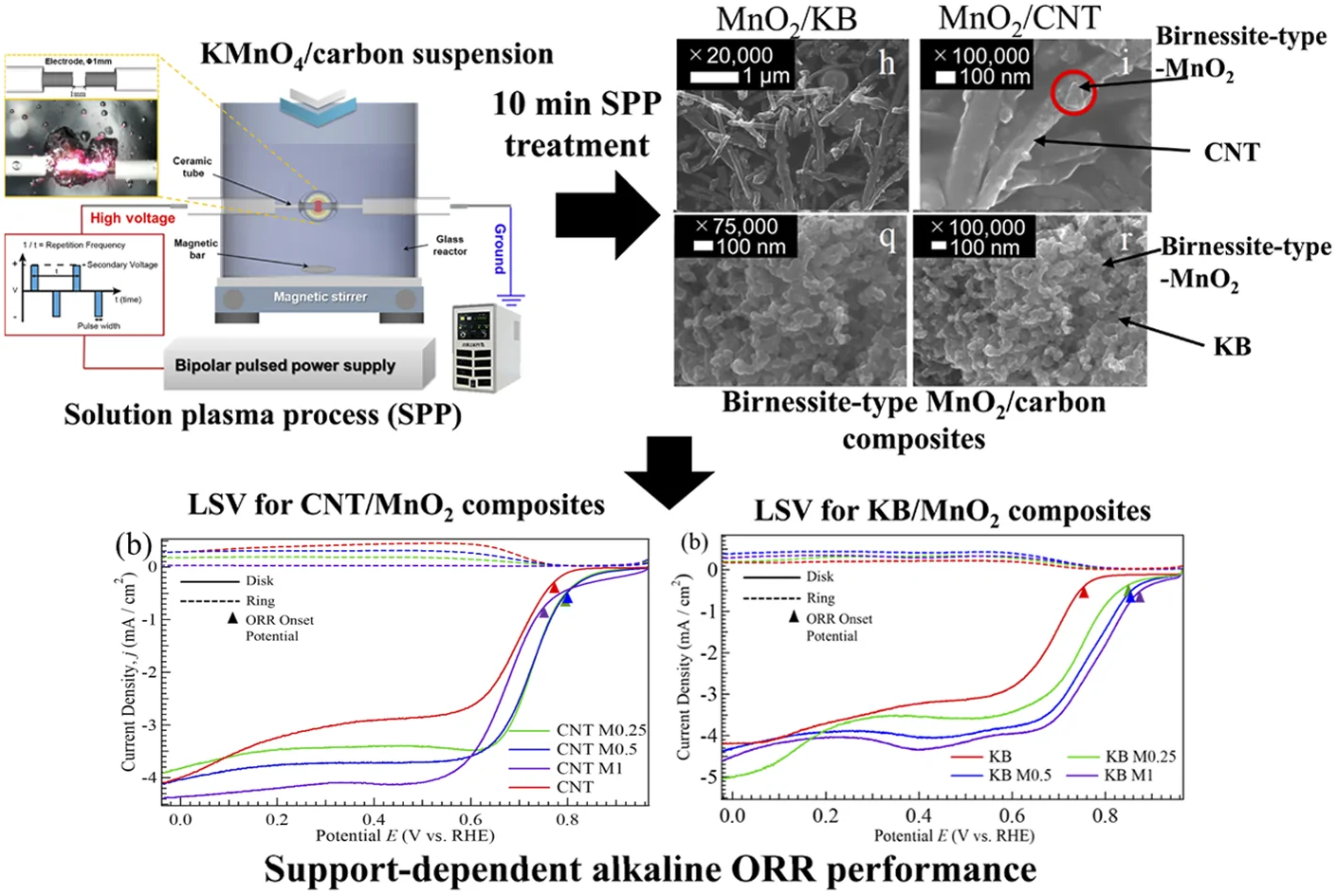

## 1. Introduction

Efficient and affordable oxygen reduction reaction (ORR) electrocatalysts are required for alkaline oxygen electrodes, including those used in zinc–air batteries. Non-precious-metal oxides and carbon-based materials have attracted attention as alternatives to Pt-based catalysts.^1–5^ Related manganese-oxide oxygen electrodes have also been investigated for zinc–air batteries.^6^ Developing these alternatives requires consideration of both the catalytic properties of the active phase and its integration into a conductive, electrolyte-accessible electrode structure. Consequently, synthesis strategies that combine oxide formation with deposition on carbon supports are particularly relevant to the design of practical oxygen electrocatalysts. The present work evaluates the ORR in aqueous 0.1 M KOH and therefore addresses alkaline oxygen electrocatalysis.

Manganese oxides are attractive because of their abundance, cost, and tunable local coordination and oxidation states.^7,8^ Their ORR response depends on crystal structure, morphology, hydration, interlayer species, defects, and electrical transport.^6,9–19^ Among these materials, birnessite-type δ-MnO_2_ has a layered structure that accommodates interlayer species and structural disorder, providing a useful platform for investigating how local chemical environments are associated with oxygen electrocatalysis.^17,18^ However, its electrode performance also depends on the accessibility of the oxide surface and electrical connectivity within the composite. These considerations motivate the integration of manganese oxides with conductive carbon supports rather than optimization of the oxide phase alone.^7,17,20–22^ Mixed Mn valence and defect chemistry can influence oxygen-intermediate adsorption.^5,14,23^ However, a fitted Mn^3+^ fraction alone does not uniquely determine activity or establish the presence of oxygen vacancies. The relationship between Mn spectral response and ORR behavior must therefore be considered together with oxide deposition, support characteristics, and electrode accessibility.

Carbon supports provide electrical pathways and surfaces for oxide deposition.^20–22,24–26^ Their role extends beyond electrical conduction: differences in surface chemistry and aggregate structure can affect where oxide deposition occurs and how the resulting oxide is exposed to the electrolyte. Studies of metal-oxide/CNT composites and mixed oxides supported on oxidized CNTs demonstrate the relevance of combining oxide chemistry with carbon architecture in alkaline oxygen electrocatalysis.^27,28^ More recent studies of plasma-treated Co/CoO-containing carbon architectures, manganese–cobalt oxides supported on modified nanocarbons, and multicomponent heterostructured catalysts further illustrate the scope for improving oxygen-electrode performance through coordinated design of the active phase and its surrounding structure.^29–31^ CNTs and KB differ in graphitic organization, porosity, specific surface area, and aggregate structure. Nitric acid pretreatment was required to improve CNT dispersibility in the aqueous precursor solution, whereas KB could be dispersed without this treatment. This practical difference in preparation also introduces a surface-chemistry variable that must be considered alongside the differences in support architecture and surface area. These differences make CNTs and KB informative contrasting supports for investigating oxide/carbon composite formation. At the same time, they require the observed trends to be interpreted as support-dependent behavior arising from the combined effects of structure and surface chemistry.

Conventional oxide/carbon synthesis routes include permanganate-driven redox deposition, reflux, and hydrothermal processing.^7,17,20–22^ These approaches provide established routes to manganese-oxide composites, but a complementary strategy is to generate a reactive environment directly within a suspension containing both the oxide precursor and carbon support. Such an approach is attractive when oxide formation and deposition can be achieved within a short processing period, allowing precursor concentration and support selection to be explored without changing the basic synthesis procedure.

Solution plasma process (SPP) provides an alternative reaction environment without external heating. In SPP processing, electrical discharge within a liquid generates a localized reactive environment through interactions among energetic electrons, excited species, and the surrounding medium. Studies using aqueous permanganate have demonstrated that SPP can facilitate manganese-oxide formation on a timescale of minutes.^32^ For oxide/carbon composite preparation, this offers the possibility of bringing precursor conversion and deposition onto dispersed carbon surfaces into the same processing step. The aqueous medium and operation at atmospheric pressure also make SPP an accessible alternative to synthesis requiring a heated, pressurized reaction vessel. In the present study, the oxide/carbon formation step uses 10 min of SPP treatment without external heating, providing a concise route for preparing a comparative series of composites. These practical features motivate the use of SPP here, although a quantitative assessment of energy consumption would require separate measurements.

SPP has also been used to modify carbon-based materials.^33^ It is therefore relevant to oxide/carbon synthesis not only as a means of precursor conversion, but also as a processing environment in which the carbon surface may change during composite formation. This possibility creates an opportunity to examine oxide deposition and carbon surface chemistry together, rather than treating the support as an unchanged substrate. Reactive species generated during SPP may contribute to precursor conversion and interface formation, although their identities and relative contributions are not measured in the present carbon-containing suspensions. Moreover, permanganate can react spontaneously with carbon materials, as exploited in established manganese-oxide deposition methods.^20,21^ Accordingly, the composites obtained here are described as solution plasma-processed materials, while the individual contributions of discharge-driven chemistry and permanganate–carbon reactions remain to be resolved.

Previous studies have already established SPP-based routes to MnO_2_–carbon hybrids. Kim and co-workers reported a one-pot plasma-discharge synthesis of MnO_2_-carbon nanoparticle composites and investigated their ORR and oxygen evolution reaction activity.^34^ Pimklang and co-workers subsequently prepared graphene oxide-MnO_2_ composites by SPP for energy-storage applications.^35^ These reports establish the feasibility of SPP-assisted oxide/carbon formation and provide a basis for examining how the choice of a pre-existing carbon support affects the resulting composite. Building on this foundation, the present work focuses on the comparative question of how contrasting support characteristics and precursor concentrations are associated with manganese-oxide deposition, surface-spectroscopic response, and alkaline ORR behavior under a common SPP protocol.

Here, we compare CNT- and KB-supported birnessite-type manganese oxide composites prepared by 10 min SPP at three KMnO_4_ concentrations. Structural, surface-spectroscopic, and electrochemical measurements are used to examine associations among oxide deposition, carbon surface chemistry, Mn spectral response, and alkaline ORR performance. The contribution of this study lies in combining two contrasting carbon supports with a precursor-concentration series, thereby examining how the response to increased precursor concentration depends on the support. Evaluating ORR activity, peroxide selectivity, and current retention alongside structural and surface measurements provides a broader assessment than comparison of a single activity descriptor. This approach helps identify the balance among oxide formation, accessible electrode structure, and electrochemical performance that is relevant to the design of SPP-based manganese-oxide/carbon catalysts. Possible interfacial and oxide-growth contributions are discussed with explicit recognition of the limits of the experimental design.

## 2 Experimental methods

### 2.1 Preparation of MnO_2_/Carbon composite precursor solutions

To prepare the MnO_2_/carbon composite precursors, potassium permanganate (KMnO_4_, Kanto Chemical Co., Ltd) was dissolved in ultrapure water to create solutions at concentrations of 0.25, 0.5, and 1.0 mM. Subsequently, 25 mg of the respective carbon support—either Ketjenblack (KB: resistivity ≈3.9 Ω cm, specific surface area ≈1350 m^2^ g^−1^) or hydrophilic Cup Stacked Carbon Nanotubes (CSCNT, GSI Creos Co., Ltd: resistivity ≈0.04 Ω cm, specific surface area ≈38 m^2^ g^−1^)—was dispersed in the KMnO_4_ solution. Before composite preparation, the CSCNT was subjected to acid treatment to enhance its hydrophilicity and dispersibility. For this procedure, 0.3 g of CSCNT was added to 70 mL of a 3 M nitric acid (HNO_3_) aqueous solution. The suspension was stirred at 1000 rpm and 80 °C for 20 minutes. This was followed by ultrasonic treatment for 2 h. The resulting treated CSCNT was collected by filtration, washed thoroughly with ultrapure water to neutral pH, and dried in an oven at 150 °C for 4 h to obtain the hydrophilic support material.

The as-received CNTs did not disperse adequately in the aqueous KMnO_4_ precursor solution under the preparation conditions used in this study. Nitric acid treatment was therefore applied to modify the CNT surface and improve its aqueous dispersibility. In contrast, KB dispersed in the aqueous precursor solution without nitric acid treatment and was used as received. Accordingly, the pre-synthesis CNT reference used for XPS, TEM, and Raman characterization represents the nitric-acid-treated CNT support before SPP, whereas the KB reference represents the untreated support. Although the different pretreatment procedures were selected to obtain aqueous dispersions suitable for composite preparation, nitric acid treatment may also alter CNT surface chemistry and subsequent manganese-oxide deposition. This asymmetric pretreatment is therefore considered together with differences in support structure and surface area when interpreting the CNT-KB comparison.

### 2.2 Synthesis of MnO_2_/carbon composite by SPP

The MnO_2_/carbon composites were synthesized in a solution plasma reactor using a pair of tungsten electrodes (W, 99.95%, Nilaco, 1.0 mm diameter). Fig. 1 shows a schematic illustration of the SPP setup. Synthesis was performed by generating plasma in the precursor solutions using a bipolar pulse power supply (MPP-HV04, Kurita Seisakusho Co., Ltd, Japan). The parameters for the SPP treatment were fixed as follows: a frequency of 100 kHz, a pulse width of 0.6 µs, and an electrode distance of 1 mm. Before SPP treatment, the tungsten electrodes were polished using #400 and #1000 abrasive paper before each run, and their sides were insulated so that only the tips were exposed. Processing was conducted at ambient pressure without external heating. Additionally, the precursor solution was stirred continuously at 600 rpm during the SPP process for 10 min.

**Fig. 1:**
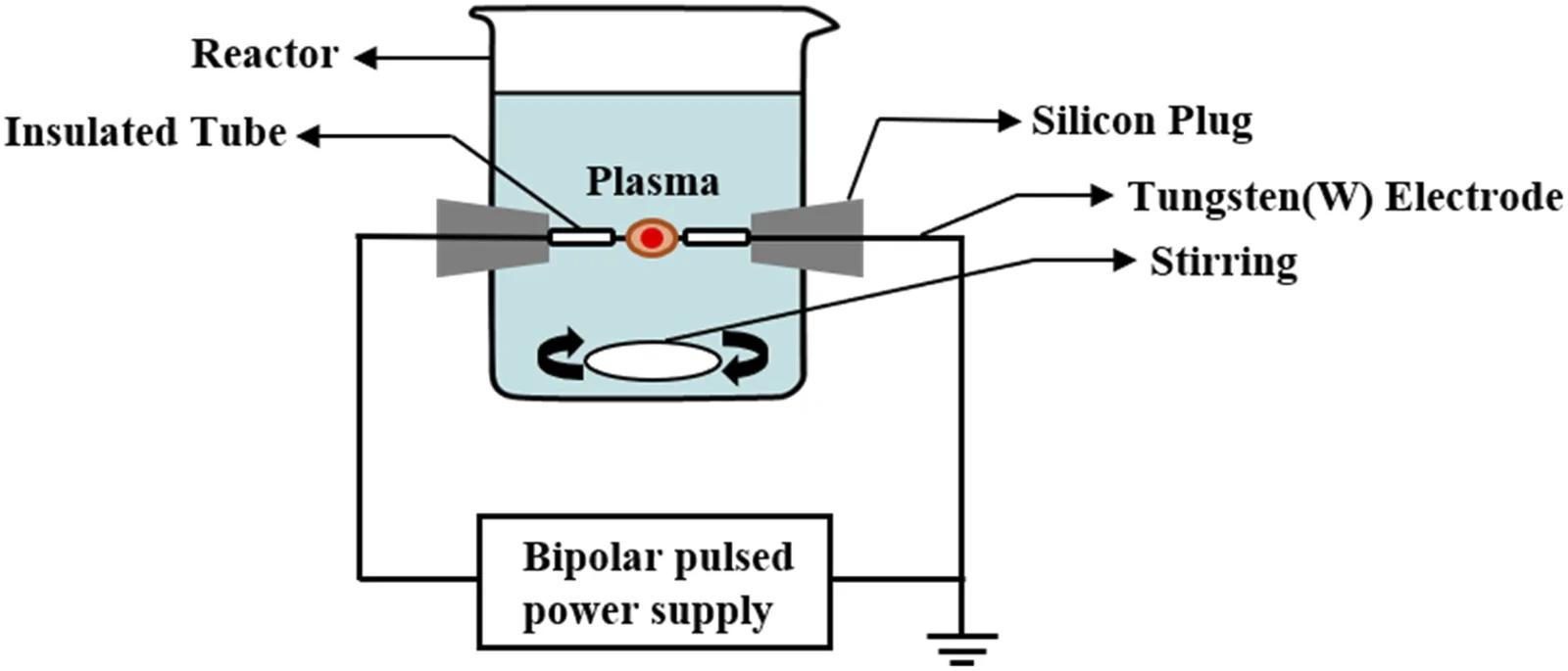
Schematic of the solution plasma reactor used for composite preparation.

Reliable optical emission spectra could not be obtained through the optically absorbing and scattering carbon-containing suspensions in the present collection geometry. Quantitative plasma temperatures and electron density were therefore not determined. Electron, excitation, gas, and liquid temperatures must be distinguished in this non-equilibrium system, and the electrical pulse settings alone do not establish these quantities. Individual reactive species discussed below are possible participants rather than species directly identified in this study.

After the SPP treatment, the synthesized samples were filtered using a hydrophilic PTFE membrane filter (JVWP04700, Merck Millipore Co., Ltd, USA) with a pore size of 0.1 µm. The recovered samples were then thoroughly washed multiple times with both ultrapure water and ethanol (purity >95.0%, Kanto Chemical Co., Inc., Japan). Following washing, the filtered samples were dried in an electric oven at 140 °C for 4 h. The resulting dried composite materials were pulverized with an agate mortar to obtain fine powder samples for subsequent analysis.

Hereafter, the samples are expressed as CNT, CNT M0.25, CNT M0.5, CNT M1, KB, KB M0.25, KB M0.5, and KB M1 based on the carbon type and the KMnO_4_ concentrations of the precursor solution.

### 2.3 Characterization

The crystal phase of the synthesized samples was identified using X-ray diffraction (XRD, Rigaku Co., Smart Lab). The measurements were conducted using Cu-Kα radiation (*λ* = 0.154 nm) operating at 40 kV and 40 mA (1.6 kW). The morphology and shape of the synthesized samples were observed using Field Emission Scanning Electron Microscopy (FE-SEM, JEOL Ltd, JSM-7100F) at an accelerating voltage of 15 kV. The atomic composition of the synthesized samples was quantitatively determined using energy-dispersive X-ray spectroscopy (EDS, JEOL Ltd, JED-2300F), which was performed at an accelerating voltage of 20 kV. Qualitative analysis of the bonding states and surface composition was performed using X-ray photoelectron spectroscopy (XPS) (JEOL Ltd, JPS-9010). XPS measurements utilized Mg-Kα radiation (1253.6 eV) as the excitation source, conducted under ultra-high vacuum conditions at a voltage of 10 kV and a current of 25 mA. Spectra were analyzed using JEOL Spec Surf 2.0.1. The C 1s reference was set to 284.6 eV for charge correction, and a Shirley background was used. Peak fitting was performed using mixed Lorentzian–Gaussian line shapes. The same number of components and component assignments were applied to the corresponding core-level spectra of all six composites. The Lorentzian/Gaussian mixing parameter and FWHM were allowed to vary among the spectra. The ranges of the fitted parameters obtained across the six composites are summarized in Table S1. Transmission electron microscopy (TEM) was performed using a JEM-2100 instrument (JEOL Ltd) at an accelerating voltage of 200 kV. Raman spectra were collected using an NRS-5100 spectrometer (JASCO Co.) with 532 nm excitation at a laser power of 0.4 mW. To examine changes in morphology and Raman spectral features following the chronoamperometry (CA) durability test, the synthesized samples before CA testing were compared with samples recovered from the electrodes after CA testing using FE-SEM and Raman spectroscopy. After the CA measurements, the catalyst-coated electrodes were rinsed in ultrapure water for 10 min while rotating at 1500 rpm, followed by drying under ambient conditions. The dried catalyst layers were then detached from the electrodes, and the recovered powders were analyzed without further treatment. The post-CA characterization therefore reflects the material remaining on the electrodes after testing and subsequent rinsing. Possible changes introduced during rinsing, drying, and powder recovery should be considered when interpreting the comparison.

### 2.4 Electrochemical measurements

The electrocatalytic performance of the synthesized composite materials for the Oxygen Reduction Reaction (ORR) was evaluated using a standard three-electrode configuration in a 0.1 M KOH aqueous solution (Kanto Chemical Co., Inc., Tokyo, Japan) at room temperature.

#### 2.4.1 Apparatus and electrodes

All electrochemical measurements were conducted using a computer-controlled ALS-CH instrument electrochemical analyzer (Model 704ES, BAS, Co.) equipped with a rotating ring disk electrode (RRDE) rotator apparatus (RRDE-3A, ALS Co.). A glassy carbon disk (GC disk: diameter 4 mm, *A*_disk_ = 0.126 cm^2^) of the RRDE, surrounded by a Pt ring (inner/outer diameter: 5.0/7.0 mm, *A*_ring_ = 0.188 cm^2^), was used as the working electrode (WE). A Pt coil (ALS, Co., Ltd) and an Ag/AgCl electrode (ALS, Co., Ltd) filled with saturated KCl aqueous solution were used as counter and reference electrodes, respectively.

#### 2.4.2 Catalyst ink preparation and working electrode fabrication

The catalyst ink was prepared by uniformly suspending 5.0 mg of the synthesized sample in a mixture of 480 µL ultrapure water, 480 µL ethanol (99.5% purity, Kanto Chemical Industries, Ltd, Tokyo, Japan), and 40 µL of a Nafion® aqueous solution (5 wt%, FUJIFILM Wako Pure Chemical Corporation, Osaka, Japan). This suspension was thoroughly sonicated until a homogeneous dispersion (approximately 5 mg mL^−1^) was achieved. Subsequently, 7.5 µL of this ink was precisely drop-cast onto the GC disk of the RRDE and allowed to dry completely in the air to form the working electrode film.

#### 2.4.3 Measurement procedure

Before measurement, the 0.1 M KOH electrolyte solution was purged with N_2_ gas for at least 30 minutes to ensure the complete removal of dissolved O_2_. The ORR catalytic performance was primarily assessed by linear sweep voltammetry (LSV). Following the N_2_ purge, the electrolyte was saturated with O_2_ gas by flowing O_2_ for 20 minutes. O_2_ gas was continuously flowed over the surface of the electrolyte throughout the LSV measurements. The LSV was performed on the disk electrode at a rotation speed of 1500 rpm and a scan rate of 10 mV s^−1^, conducted within a potential range of −0.02 to 0.96 V *vs.* RHE. The RRDE configuration was specifically utilized to calculate the number of transferred electrons (*n*) and the hydrogen peroxide (H_2_O_2_) production rate during the ORR. Cyclic Voltammetry (CV) was also employed in an O_2_-saturated electrolyte to further assess the overall catalytic performance at a scan rate of 50 mV s^−1^ and within a potential range of −0.04 to 1.16 V *vs.* RHE.

An apparent capacitance (*C*_app_) was estimated from cyclic voltammetry (CV) measurements performed at scan rates of 0.01–0.05 V s^−1^ over the potential range of 0.1–0.3 V *vs.* RHE in O_2_-saturated 0.1 M KOH. Because oxygen reduction and oxide-related faradaic/pseudocapacitive contributions may be present in this window, (*C*_app_) was not interpreted as a purely double-layer capacitance or as a direct measure of the electrochemically active surface area (ECSA). A common reference capacitance of 0.04 mF cm^−2^ was used only to define a capacitance-derived apparent area proxy. The calculation, units, and limitations of this proxy, together with its use for current normalization, are described in SI Section S1. This proxy does not directly quantify the number of Mn catalytic sites, and currents normalized using it are not interpreted as intrinsic Mn-site activities. All potentials measured against the Ag/AgCl (saturated KCl) reference electrode were converted to the reversible hydrogen electrode (RHE) scale using the Nernst equation: *E*_RHE_ = *E*_Ag/AgCl_ + 0.197 + 0.059 × pH, where *E*^0^_Ag/AgCl_ is 0.197 V at 25 °C. In this study, for 0.1 M KOH electrolyte (pH ≈ 13), the conversion is *E*_RHE_ = *E*_Ag/AgCl_ + 0.964 V.

The apparent electron-transfer number and peroxide yield were calculated from the background-corrected disk (*I*_D_) and ring currents (*I*_R_) using the following equations:1
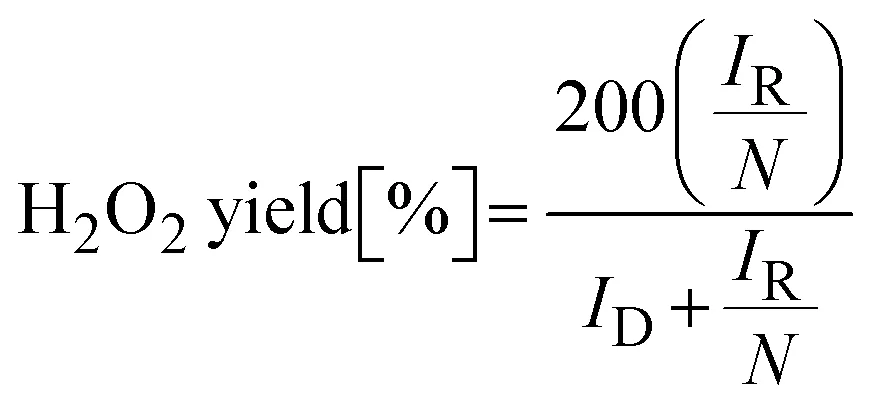
2
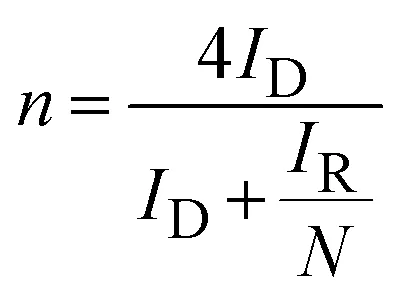
where *N* is the ring collection efficiency. The RRDE collection efficiency was taken as *N* = 0.43, based on a previous calibration using the same RRDE geometry in 0.1 M KOH containing 10 mM K_3_[Fe(CN)_6_].^36^

## 3 Results and discussion

### 3.1 Structural analysis

Fig. 2 presents the XRD patterns of the CNT/δ-MnO_2_ and KB/δ-MnO_2_ composite materials synthesized by SPP. A comparison of the bare carbon supports reveals a contrast in graphitic ordering: CNT (Fig. 2a, red) exhibits a sharp, intense (002) diffraction peak at 2*θ* ≈ 26.5°, indicative of its well-ordered graphitic structure, whereas KB (Fig. 2b, red) displays a broad, low-intensity (002) feature, consistent with a lower degree of graphitic ordering. The reference diffraction positions used for phase identification are indicated in Fig. 2. The graphitic-carbon reference positions occur near 2*θ* = 26.5° and 43° (PDF no. 65-6212), whereas the reference positions for birnessite-type δ-MnO_2_ occur near 2*θ* = 11.7°, 37.8°, and 66.0° (PDF no. 18-0802). The visibility of these features differs among the supports and composite samples because of differences in carbon ordering, manganese-oxide content, peak broadening, and overlapping contributions. The measured diffraction features near the δ-MnO_2_ reference positions are broad and relatively weak, consistent with the formation of poorly crystalline birnessite-type manganese oxide.^37^ Limited coherent domain size and structural disorder, including possible turbostratic stacking of the MnO_6_ layers, may contribute to the observed broadening. However, their individual contributions cannot be distinguished from these patterns alone. The feature near 2*θ* ≈ 37.8° exhibits the highest intensity among the δ-MnO_2_ diffraction features in the XRD patterns of both series.

**Fig. 2:**
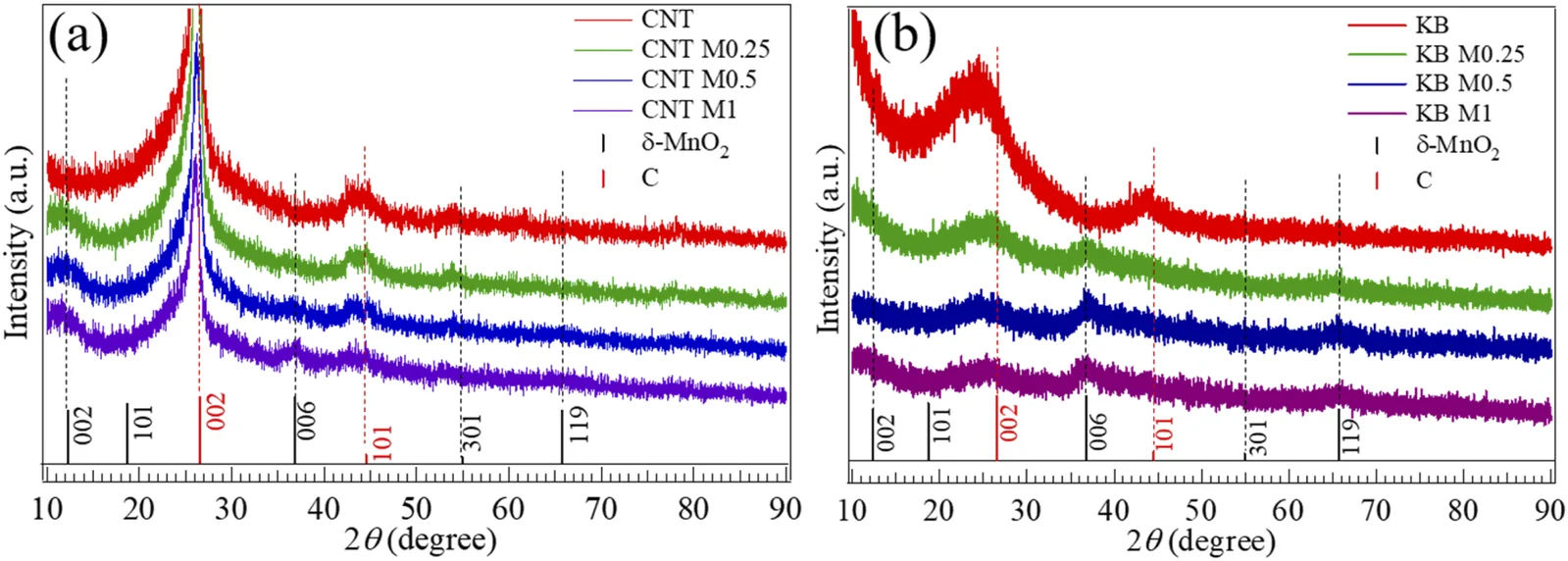
XRD patterns of the manganese oxide/carbon composites based on (a) CSCNT and (b) KB. The vertical dashed red lines indicate the principal graphitic-carbon diffraction positions near 2*θ* = 26.5° and 43° (PDF no. 65-6212). The birnessite-type δ-MnO_2_ phase (vertical dashed black lines) was identified by comparison with PDF no. 18-0802, as discussed in Section 3.1.

In the CNT-M series (Fig. 2a), the δ-MnO_2_ low-angle reflection at 2*θ* ≈ 11.7° becomes discernible from CNT-M0.25 and progressively intensifies with increasing KMnO_4_ concentration, consistent with an increased contribution from the manganese-oxide phase at higher precursor concentrations. Notably, the sharp graphitic (002) peak of CNT is retained across all composite samples, indicating that graphitic domains remain detectable after SPP. This observation does not exclude changes in the CNT surface chemistry or local defect structure, which are examined separately by XPS and Raman spectroscopy.

A notable observation in the KB-based samples (Fig. 2b) is that the carbon diffraction feature near its reference position at 2*θ* ≈ 43° is no longer clearly resolved after composite formation. This is already apparent in KB-M0.25 and persists across all KB composite samples. This behavior contrasts with the CNT series, where the corresponding carbon feature remains visible, and may reflect the intrinsically weaker and broader carbon signal of KB together with the increased contribution from manganese oxide and changes in the diffraction background. The loss of a separately resolved carbon feature does not, by itself, demonstrate destruction of the carbon framework. Additionally, the diffraction feature near the δ-MnO_2_ reference position at 2*θ* ≈ 66.0° is more distinctly resolved in the KB series than in the CNT series. However, this difference alone is insufficient to establish support-dependent preferred orientation or local structural ordering, because oxide content, peak broadening, and background contributions can also affect the apparent intensity and visibility of the feature.

Regarding the overall patterns in both series, the diffraction intensity attributed to the carbon support decreases relative to that of the birnessite-type manganese-oxide contribution as the initial KMnO_4_ concentration increases. This trend is consistent with an increased relative contribution from manganese oxide in the composites. Nevertheless, a constant SPP treatment time does not ensure identical changes in the carbon structure across different precursor concentrations, and relative diffraction intensities are influenced by phase content, crystallinity, preferred orientation, absorption, and background contributions. They therefore do not independently establish oxide surface coverage or provide a quantitative measure of manganese-oxide loading. These aspects are considered together with the microscopy and elemental-composition results. Taken together, the XRD patterns are consistent with the formation of poorly crystalline birnessite-type manganese oxide in both CNT- and KB-based composites prepared by SPP and show precursor-concentration-dependent changes in the relative contributions of the carbon and manganese-oxide diffraction features.

The small number of broad and relatively weak oxide reflections, together with the carbon contribution and possible turbostratic stacking disorder, limits the reliability of conventional Rietveld refinement based on a fully periodic three-dimensional structural model. Under these conditions, lattice parameters, crystallite-size/strain terms, peak-shape parameters, and background contributions would be strongly correlated. Rietveld refinement was therefore not performed. Accordingly, the present XRD data are used for phase assignment and qualitative comparison of the diffraction features, rather than for precise determination of lattice parameters, crystallite size, microstrain, or small support-dependent lattice distortions.

### 3.2 Morphology and elemental analyses

The morphology and elemental composition of the synthesized composites were characterized using SEM and EDS. TEM images of the carbon supports before composite synthesis are provided in Fig. S1, showing the acid-treated CNT (Fig. S1a and b) and pristine KB (Fig. S1c and d). These images provide a morphological reference for interpreting the composite microscopy results. Fig. 3 presents the SEM images, with panels (a)–(i) corresponding to the CNT-M series and panels (j)–(r) to the KB-M series. The EDS maps of Mn, O, and C for selected composites are provided in Fig. S2 for the CNT-M series and Fig. S3 for the KB-M series.

**Fig. 3:**
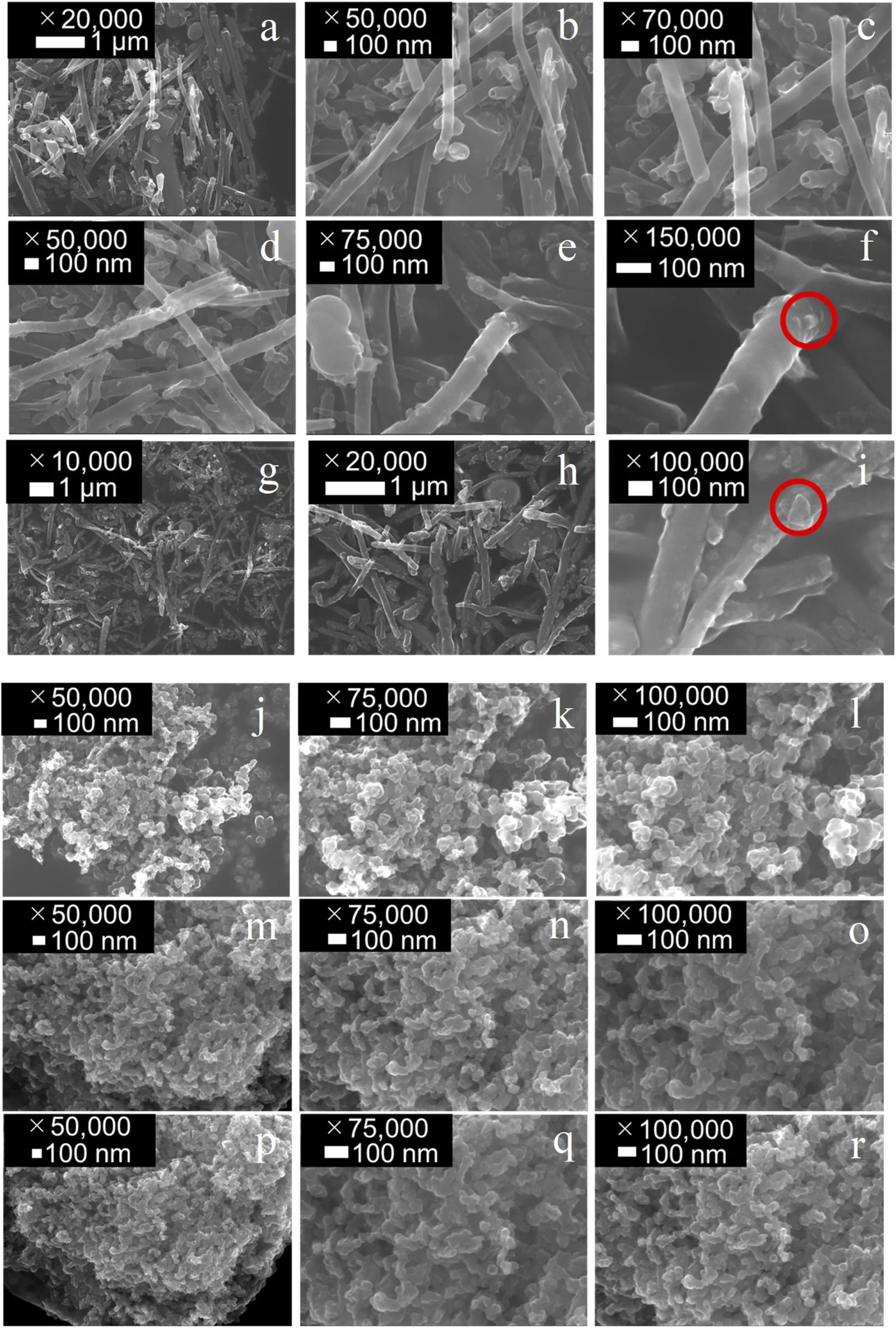
SEM images of (a–c): CNT-M0.25, (d–f): CNT-M0.5, (g–i): CNT-M1, (j–l): KB-M0.25, (m–o): KB-M0.5, (p–r): KB-M1.

#### 3.2.1 CNT/δ-MnO_2_ composites

The SEM images (Fig. 3a–i) show the tubular carbon framework and increasing oxide deposition with precursor concentration. At the lowest precursor concentration, CNT-M0.25 exhibits sparse particle deposition, with substantial regions of the CNT surface remaining exposed. More extensive particle deposition is observed in CNT-M0.5 and CNT-M1. The EDS elemental maps of Mn, O, and C (Fig. S2) complement these observations: the Mn signal is locally distributed along the CNT framework in CNT-M0.5, whereas a more extensive Mn distribution is observed in CNT-M1. These maps support the association of the deposited material with manganese oxide, although they do not quantitatively establish surface coverage, coating thickness, or uniformity. The oxide-associated particle sizes are estimated to be approximately 20–30 nm in CNT-M0.5 and 50–100 nm in CNT-M1. These are approximate image-based size estimates rather than a statistically resolved particle-size distribution. Particle coarsening and aggregation may reduce the exposed surface-to-volume ratio and obstruct electrolyte access to carbon/oxide regions. This is consistent with the decrease in the capacitance-derived area proxy from CNT-M0.5 to CNT-M1 despite increasing EDS Mn content, although the capacitance-derived proxy does not independently establish the cause of this decrease. Size-dependent changes in surface and near-surface Mn environments may also contribute to the XPS response, although the direction of this effect is not established. The SEM sizes do not determine oxide layer thickness or the number of birnessite layers.

#### 3.2.2 KB/δ-MnO_2_ composites

The KB-M samples exhibit an aggregated carbon morphology (Fig. 3j–r). Individual oxide particles are less readily resolved than in the CNT-M series, and differences in aggregate surface texture are observed across the precursor-concentration series. The EDS elemental maps (Fig. S3) show Mn signals distributed across the sampled KB aggregates, with a more extensive signal in KB-M1. Together with the increasing EDS Mn atomic fraction (Table 1), these observations are consistent with manganese-oxide deposition on the KB-based material. The large quoted surface area and aggregate structure of KB may contribute to the deposition behavior, alongside surface chemistry and other preparation variables. However, the images do not quantify coating thickness or establish conformal coverage throughout the internal pore network, and they do not identify surface defects as the independent cause of deposition.

**Table 1:** Atomic concentrations of CNT-M0.25, CNT-M0.5, CNT-M1, KB-M0.25, KB-M0.5, and KB-M1 obtained by EDS

(at.%)	C	O	Mn	K
CNT-M0.25	98.8	0.93	0.02	Not detected
CNT-M0.5	98.8	0.88	0.17	Not detected
CNT-M1	97.8	1.17	0.37	Not detected
KB-M0.25	97.7	1.49	0.51	0.14
KB-M0.5	95.6	2.32	1.32	0.45
KB-M1	87.5	5.67	4.13	1.64

#### 3.2.3 EDS quantitative analysis

The EDS Mn atomic fraction increases with precursor concentration in both series. At M1, the values are 4.13 at% for KB-M1 and 0.37 at% for CNT-M1, a ratio of approximately 11.2. The quoted support surface areas, 1350 and 38 m^2^ g^−1^, differ by approximately 35.5-fold. The larger available area and porous aggregate structure of KB may favor more extensive oxide deposition and dispersion. However, EDS atomic fractions are local composition measurements, not bulk Mn mass loadings or deposition yields. These data do not quantitatively separate surface-area, surface-chemistry, pretreatment, and morphology effects.

Potassium is detected in the KB composites. Its presence is compatible with potassium-containing birnessite, but EDS does not locate K within the interlayer or exclude residual surface potassium species. The lower oxide content in the CNT composites may contribute to the absence of a detectable K signal. Absolute loading and incorporation-site assignments would require additional bulk and structure-sensitive measurements.

#### 3.2.4 XPS analysis

XPS analysis was conducted to examine the surface elemental composition and chemical states of the six composite samples. The O 1s and Mn 2p core-level spectra are presented in Fig. 4 and 5, respectively, and the C 1s spectra are provided in Fig. S4. The corresponding O 1s and C 1s spectra of the pre-synthesis supports are also shown in Fig. S5 and S6, respectively. The fitted component fractions are therefore comparisons within a common fitting model and assignment procedure rather than model-independent measurements of absolute chemical-state populations.

**Fig. 4:**
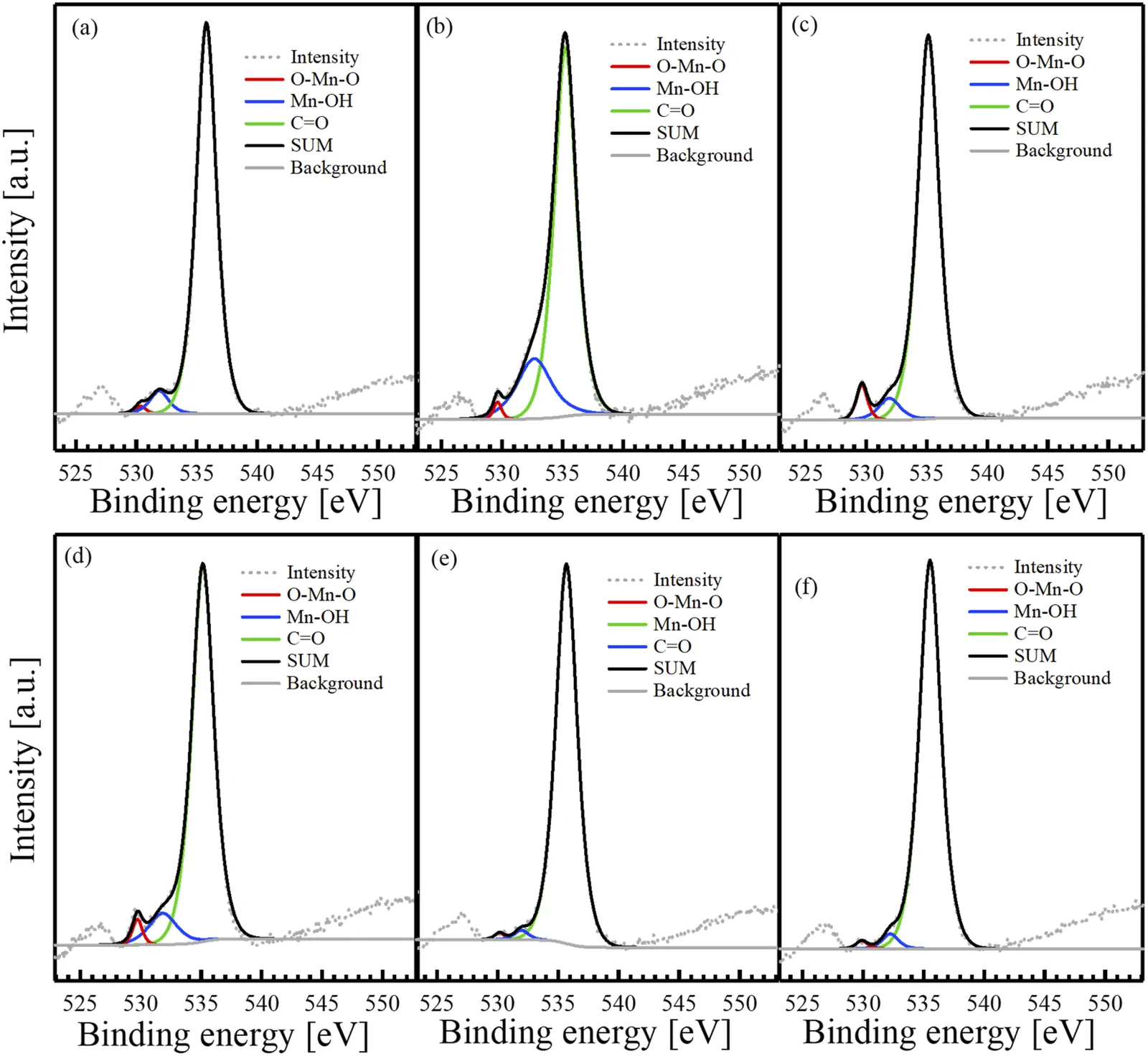
XPS O 1s spectra of (a) CNT-M0.25, (b) CNT-M0.5, (c) CNT-M1, (d) KB-M0.25, (e) KB-M0.5, and (f) KB-M1. The fitted components are assigned to O–Mn–O lattice oxygen, Mn–OH/hydroxyl-related oxygen, and carbonyl-related oxygen (C=O).

**Fig. 5:**
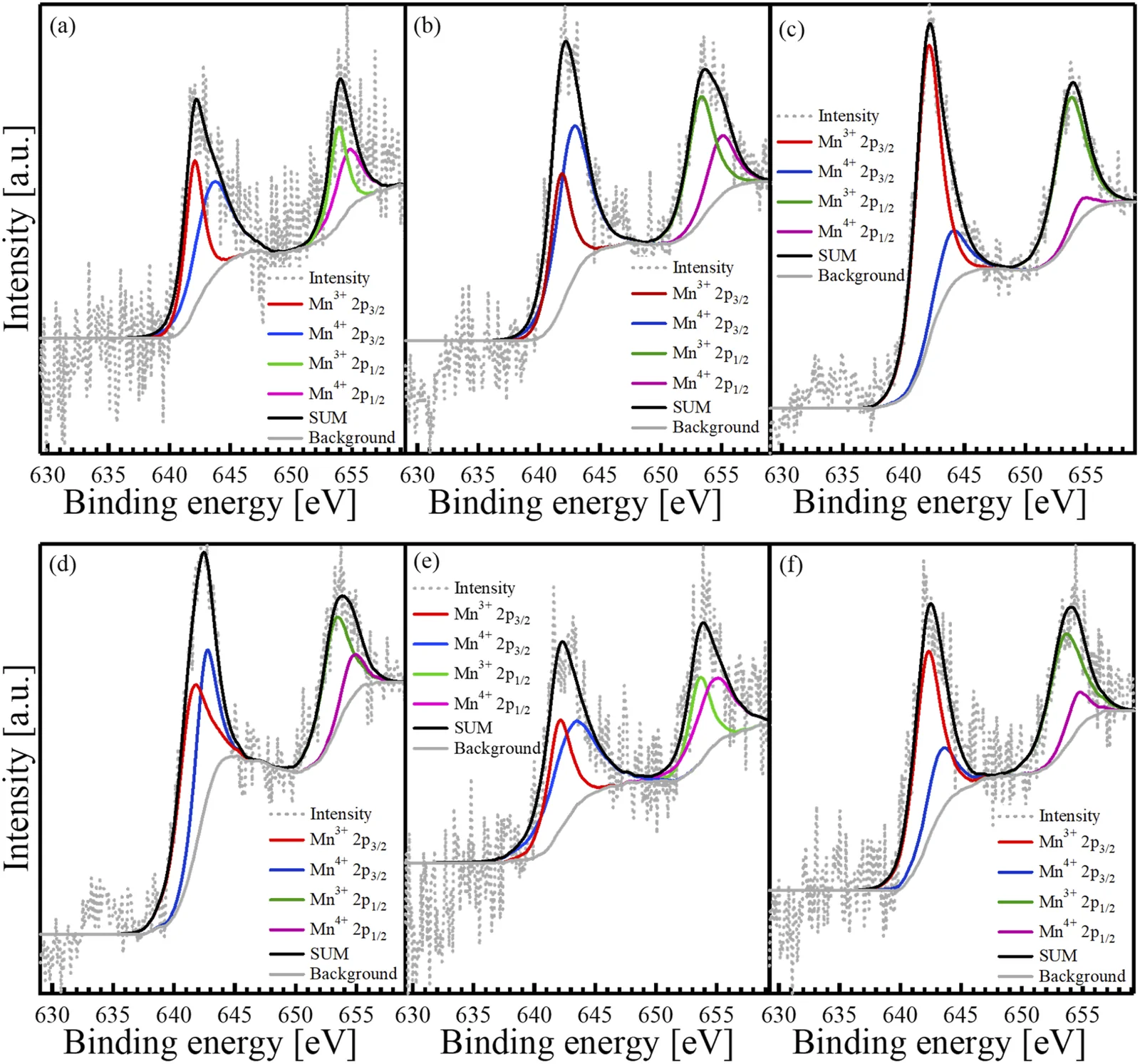
XPS Mn 2p spectra of (a) CNT M0.25, (b) CNT M0.5, (c) CNT M1, (d) KB M0.25, (e) KB M0.5, and (f) KB M1.

The O 1s spectra of all six composites (Fig. 4) were fitted using three components at approximately 530.0, 531.4, and 535.2 eV. The components near 530.0 and 531.4 eV were assigned to O–Mn–O lattice oxygen and Mn–OH/hydroxyl-related oxygen, respectively.^38^ Within the fitting model adopted in this study, the high-binding-energy component near 535.2 eV was tentatively assigned to a carbonyl-related oxygen contribution. However, because this binding-energy region may also contain contributions from adsorbed water or other weakly bound surface oxygen species, the assignment is treated as provisional rather than as a unique chemical identification.

Differences in the relative contributions of these components are observed across the precursor-concentration series. In the CNT-M series, the high-binding-energy component is dominant in CNT-M0.25 (Fig. 4a), for which EDS indicates a relatively low Mn content. The O–Mn–O and Mn–OH/hydroxyl-related contributions become more evident in CNT-M0.5 and CNT-M1 (Fig. 4b and c), together with the increased manganese-oxide contribution indicated by XRD and EDS. In the KB-M series, the two lower-binding-energy components are resolved from KB-M0.25 onward (Fig. 4d–f), consistent with the detectable Mn contents of the KB-based composites at all precursor concentrations (Table 1).

The O–Mn–O component at approximately 530.0 eV supports the formation of manganese oxide on both carbon supports. However, this component alone does not distinguish the δ-MnO_2_ polymorph; the assignment of the poorly crystalline birnessite-type phase is based primarily on the XRD results. The component at approximately 531.4 eV is consistent with Mn–OH and other hydroxyl-related surface species.^38^ Although hydroxylated and hydrated environments are characteristic of birnessite-type manganese oxides, neither this component nor the broad XRD reflections alone determines the amount or location of interlayer water.

The pre-synthesis O 1s spectra of the nitric-acid-treated CNT and untreated KB supports are shown in Fig. S5. These spectra demonstrate that oxygen-containing surface species were already present before composite synthesis. Consequently, the hydroxyl-related and high-binding-energy contributions observed in the composite spectra cannot be attributed exclusively to SPP. Because an untreated-CNT reference was not measured, the present comparison also does not separately quantify the effect of nitric acid treatment. Furthermore, the pre-synthesis support spectra are not carbon-only samples subjected to SPP and therefore do not independently distinguish discharge-induced changes from changes associated with permanganate reaction and manganese-oxide deposition.

The Mn 2p spectra (Fig. 5) display the characteristic 2p_3/2_ and 2p_1/2_ spin–orbit regions. The Mn 2p spectra (Fig. 5) display the characteristic 2p_3/2_ and 2p_1/2_ spin–orbit regions. The envelopes were fitted using components assigned to Mn^3+^ 2p_3/2_ (approximately 642.3 eV), Mn^4+^ 2p_3/2_ (approximately 643.25 eV), Mn^3+^ 2p_1/2_ (approximately 653.8 eV), and Mn^4+^ 2p_1/2_ (approximately 654.8 eV).^38^ The fitted Mn^3+^ and Mn^4+^ fractions were calculated by summing the corresponding 2p_3/2_ and 2p_1/2_ component areas and are summarized in Table 2.

**Table 2:** Ratios of Mn^3+^ and Mn^4+^ components derived from the deconvolution of Mn 2p XPS spectra for δ-MnO_2_-carbon composites

	Mn^3+^ 2p_3/2_ (%)	Mn^4+^ 2p_3/2_ (%)	Mn^3+^ 2p_1/2_ (%)	Mn^4+^ 2p_1/2_ (%)
CNT M0.25	29.1	36.2	17.5	17
CNT M0.5	22.1	39	26.1	12.8
CNT M1	54.7	11.6	30.4	3.15
KB M0.25	41	25.7	25.6	7.41
KB M0.5	23.7	36.4	16.4	23.4
KB M1	44	16	32.8	7.09

Within this fitting model, the CNT-M series gives Mn^3+^ fractions of 46.6% for CNT-M0.25, 48.2% for CNT-M0.5, and 85.1% for CNT-M1. The fitted contribution is therefore similar for CNT-M0.25 and CNT-M0.5 and larger for CNT-M1. The KB-M series gives Mn^3+^ fractions of 66.6%, 40.1%, and 76.8% for KB-M0.25, KB-M0.5, and KB-M1, respectively, showing a non-monotonic concentration dependence. At the matched precursor concentrations, KB-M0.25 has a larger fitted Mn^3+^ fraction than CNT-M0.25, whereas the CNT value is larger at M0.5 and M1. Thus, the present results do not show an overall tendency for KB to possess a higher Mn^3+^ fraction than CNT.

The fitted spectral differences may reflect several coupled variables, including oxide loading, layer thickness, hydration, interlayer composition, defect populations, particle aggregation, and the fraction of oxide located near the carbon interface. The two supports also differ in architecture, surface area, graphitic organization, surface chemistry, and pretreatment. The present experimental design therefore does not identify carbon morphology as the independent cause of the Mn spectral differences. Interfacial electron redistribution is one possible contribution, but its direction and magnitude were not directly measured. Similarly, curvature-induced lattice strain, defect-mediated charge transfer, and oxygen-vacancy formation are not independently demonstrated by the present XPS, XRD, or microscopy data. In particular, mixed Mn valence does not uniquely establish oxygen vacancies because charge compensation can also involve interlayer cations, hydroxylation, hydration, and other structural defects.

Electronic descriptors developed for transition-metal oxides provide useful context for discussing oxygen-intermediate adsorption.^23^ In particular, *e*_g_ occupation has been related to ORR activity in well-defined transition-metal oxides, and Mn^3+^ has a different electronic configuration from Mn^4+^.^23,39^ However, quantitative transfer of this descriptor to the present poorly crystalline, carbon-supported birnessite-type materials requires caution. The electrochemical response is not determined by a single fitted Mn^3+^ fraction. For example, CNT-M1 has the largest fitted Mn^3+^ fraction in the CNT series but a lower onset potential than CNT-M0.5, while the fitted fraction in the KB series is non-monotonic even though its onset potential increases with precursor concentration. Accordingly, the Mn 2p results are discussed as support- and concentration-dependent spectral associations rather than proof of a unique Mn^3+^-controlled activity mechanism.

The C 1s core-level spectra of all six composite samples are shown in Fig. S4. The spectra were fitted using three components: a main carbon contribution near 284.6–284.7 eV, a carbonyl-related contribution near 287.5 eV, and a low-binding-energy contribution near 282.0–282.5 eV. The corresponding fitted component areas are summarized in Table S2. The main contribution near 284.6–284.7 eV is assigned to C–C/C=C bonding. Its persistence after composite preparation indicates that the carbon framework remains detectable, but does not demonstrate that the carbon structure or surface chemistry remains unchanged during SPP.

The contribution near 287.5 eV is assigned to carbonyl-related carbon species (C=O). The pre-synthesis O 1s and C 1s spectra of the nitric-acid-treated CNT and untreated KB supports are shown in Fig. S5 and S6, respectively, and demonstrate that oxygen-containing surface species were already present before composite synthesis. The carbonyl-related contribution in the composite spectra therefore cannot be attributed exclusively to SPP-induced functionalization. In particular, the pre-synthesis CNT reference represents the nitric-acid-treated support, whereas KB was used without nitric acid treatment. Differences in this contribution between the CNT- and KB-based composites may therefore reflect the combined effects of initial support surface chemistry, different pretreatment histories, and composite formation. The fitted carbonyl-related area is not treated as a direct quantitative measure of carbon defect density or oxide-carbon interfacial bonding.

The contribution near 282.0–282.5 eV is described as a low-binding-energy contribution of uncertain origin. Although low-binding-energy C 1s features have sometimes been associated with metal–carbon or carbide-like environments, the binding energy alone is insufficient to establish direct Mn–C bonding in the present composites. The Mn 2p spectra do not provide independent evidence for metallic Mn or a carbide-like low-valence Mn species. Possible effects associated with carbon surface chemistry, residual potassium-containing species, energy referencing, and differential charging cannot be distinguished using the present data; none of these possibilities is assigned as the origin of the fitted component. Accordingly, the component is not assigned uniquely to Mn–C or Mn–O–C bonding and is not used as evidence for a specific SPP-generated interfacial structure or charge-transfer pathway. The fitted component areas in Table S2 are interpreted as model-dependent spectral quantities rather than absolute concentrations of specific surface bonds.

The carbon structures were further examined using Raman spectra of the actual pre-synthesis supports and representative composites (Fig. S7). Both support types exhibit D and G bands. Their relative intensities and widths are compared using the same acquisition and analysis procedure rather than substituting literature D/G ratios for measurements of the present samples. Previous studies of related CNT and carbon-black materials provide useful context for differences in graphitic organization and disorder.^40,41^ Together with XRD, the Raman spectra support structural differences between the two carbon supports, but they do not demonstrate strain-specific behavior or the strength of electronic coupling at the oxide/carbon interface.

Overall, XPS shows support- and precursor-concentration-dependent differences in the surface oxygen, manganese, and carbon spectral responses. Combined with XRD and EDS, the spectra support manganese-oxide formation on both carbon supports. However, they do not by themselves establish direct Mn–C bonding, quantify oxygen-vacancy concentrations, isolate a morphology-only effect, or identify a single interfacial charge-transfer mechanism governing ORR activity.

### 3.3 Electrochemical ORR performance

#### 3.3.1 CNT/δ-MnO_2_ composites

The electrocatalytic ORR performance of the CNT/δ-MnO_2_ composites was evaluated by cyclic voltammetry (CV) and linear sweep voltammetry (LSV) using a rotating ring–disk electrode (RRDE) in 0.1 M KOH. The CV and LSV results are presented in Fig. 6a and b, respectively, and the LSV response of commercial 20 wt% Pt/C measured under the same electrolyte conditions is included in Fig. S9 for comparison. The oxygen-dependent current was obtained by subtracting the background current measured in N_2_-saturated electrolyte (*I*_N_2__) from the current measured in O_2_-saturated electrolyte (*I*_O_2__):

3 *I*_O_2_-dependent_ = *I*_O_2__ − *I*_N_2__

**Fig. 6:**
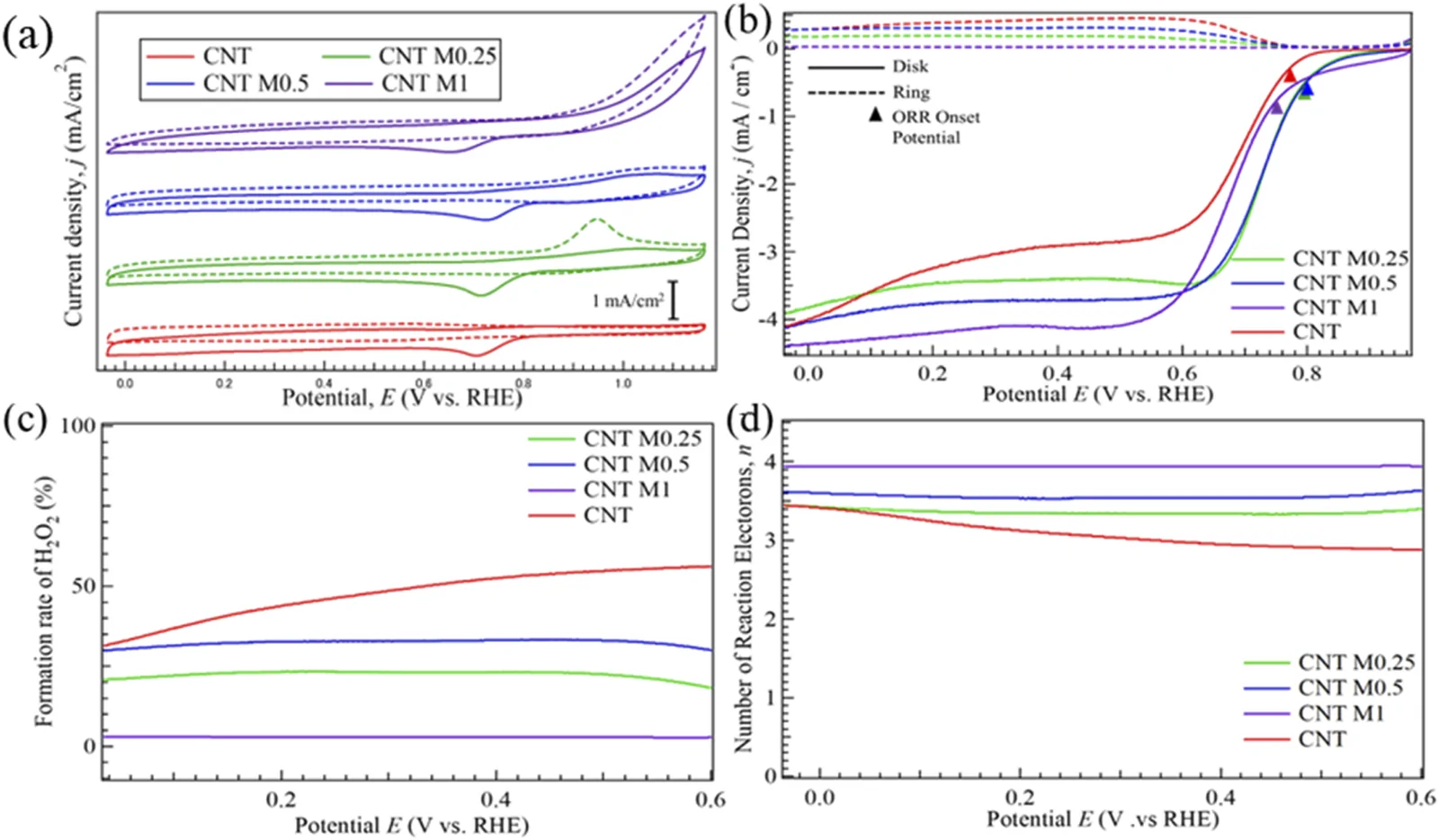
Electrochemical evaluation of CNT, CNT-M0.25, CNT-M0.5, and CNT-M1 in 0.1 M KOH: (a) CV curves recorded in N_2_- and O_2_-saturated electrolytes at 50 mV s^−1^, where the dashed and solid lines represent the responses measured under N_2_ and O_2_, respectively; (b) background-corrected RRDE polarization curves recorded in O_2_-saturated electrolyte at 10 mV s^−1^ and 1500 rpm, where the solid and dashed lines represent the disk and ring responses, respectively; (c) HO_2_^−^ yield; and (d) electron-transfer number, *n*, calculated from the disk and ring currents using *N* = 0.43. The ring potential was fixed at 0.5 V *vs.* RHE.

The N_2_-saturated response is treated operationally as the background current under the stated measurement conditions. It may include double-layer charging and carbon- and manganese-oxide-related faradaic or pseudocapacitive contributions and is therefore not assumed to be purely non-faradaic. This subtraction provides an operational estimate of the oxygen-dependent current; any oxygen-induced changes in oxide redox behavior may also contribute to the difference.

As shown in Fig. 6a, the CNT-M samples exhibit larger cathodic responses in O_2_-saturated electrolyte than in N_2_-saturated electrolyte, consistent with an oxygen-dependent reduction contribution. Redox features are also observed in the N_2_-saturated curves of the MnO_2_-containing electrodes but are absent or substantially weaker for the CNT support. Manganese oxides can undergo coupled Mn redox and proton-transfer reactions in alkaline media, and MnOOH-type surface intermediates have been proposed in related ORR systems.^41–43^ A simplified surface-redox step may be represented schematically as

4 ≡Mn(iv) − O + H_2_O + e^−^ ⇌ ≡Mn(iii) − OH + OH^−^

This equation is used to indicate a possible surface or near-surface redox step and does not establish bulk conversion of δ-MnO_2_ or the formation of a stoichiometric MnOOH phase in the present electrodes. The shapes and positions of the redox features differ among CNT-M0.25, CNT-M0.5, and CNT-M1. These differences may reflect the combined effects of manganese-oxide loading, local structure, hydration, interlayer species, particle aggregation, and electrode transport.^18,38,41–44^ The present CV data do not uniquely separate these contributions or establish a particular Mn^3+^/Mn^4+^ couple as the sole origin of each peak. Likewise, the difference between the N_2_- and O_2_-saturated anodic responses indicates that the electrode response depends on the gas environment, but it does not by itself demonstrate chemical oxidation of a specific Mn valence state by dissolved O_2_.

The LSV curves in Fig. 6b show that incorporation of manganese oxide changes both the onset potential and the geometric current density relative to the CNT support. The onset potentials of CNT, CNT-M0.25, CNT-M0.5, and CNT-M1 are 0.771, 0.799, 0.804, and 0.754 V *vs.* RHE, respectively (Table 3). CNT-M0.5 therefore exhibits the highest measured onset potential within the CNT series. At 0.464 V *vs.* RHE, the corresponding geometric current densities are −3.19, −3.46, −3.75, and −4.17 mA cm^−2^, with CNT-M1 showing the largest current magnitude. Thus, the precursor concentration giving the highest onset potential is not the same as that giving the largest current magnitude. This behavior is discussed as a loading-dependent association rather than being assigned uniquely to electrical resistance, Mn valence, or the number of Mn active sites. The CNT-M samples remain lower in onset potential than Pt/C under the present conditions (Fig. S9). The electron-transfer number, *n*, and peroxide yield calculated from the disk and ring currents according to the RRDE procedure described in Section 2.4.3 are shown in Fig. 6c and d, respectively.

**Table 3:** ORR onset potentials and RRDE parameters of CNT, CNT-M0.25, CNT-M0.5, and CNT-M1^a^

	ORR onset potential (V)	Electron transfer numbers	Peroxide yield, HO_2_^−^ (%)	Current density (mA cm^−2^)
CNT	0.771	2.92	54.0	−3.19
CNT-M0.25	0.799	3.54	22.9	−3.46
CNT-M0.5	0.804	3.53	23.5	−3.75
CNT-M1	0.754	3.94	2.86	−4.17

a Electron-transfer number, HO_2_^−^ yield, and geometric current density were evaluated at 0.464 V *vs.* RHE. The onset potential is an independently determined characteristic.

At 0.464 V *vs.* RHE, *n* increases from 2.92 for CNT to 3.54, 3.53, and 3.94 for CNT-M0.25, CNT-M0.5, and CNT-M1, respectively, while the corresponding peroxide yields decrease from 54.0% to 22.9%, 23.5%, and 2.86% (Table 3). These paired values are consistent with an increasing apparent contribution from the four-electron pathway as the manganese-oxide content increases. The particularly high *n* value and low peroxide yield of CNT-M1 indicate predominantly four-electron ORR under the specified RRDE conditions. They do not, however, identify a unique molecular pathway or demonstrate that the selectivity is controlled solely by the fitted Mn^3+^ fraction. Sequential peroxide formation and subsequent reduction or disproportionation cannot be distinguished from a direct four-electron pathway using the present RRDE data alone.^45^

The increasing geometric current magnitude and four-electron selectivity from CNT to CNT-M1 occur together with increasing Mn content determined by EDS (Table 1), but the onset-potential trend is non-monotonic. SEM observations indicate greater oxide-associated deposition and coarser aggregates at the highest precursor concentration (Fig. 3g–i). These structural and compositional changes may affect the number and accessibility of reaction sites, local mass transport, and electrical contact. Because film conductivity, charge-transfer resistance, oxide layer thickness, and Mn-site density were not independently measured, the lower onset potential of CNT-M1 than CNT-M0.5 is not assigned to a single resistance or electronic-structure mechanism.

#### 3.3.2 KB/δ-MnO_2_ composites

The electrocatalytic ORR performance of the KB/δ-MnO_2_ composites was evaluated under the same electrolyte and RRDE conditions. The CV and LSV curves are presented in Fig. 7a and b, respectively, and the Pt/C comparison is shown in Fig. S9. The KB-M electrodes display oxygen-dependent cathodic currents together with redox features whose shapes and intensities differ from those of the KB support and CNT-M electrodes. These features are consistent with overlapping ORR, manganese-oxide redox, and pseudocapacitive contributions.^18,19,41–45^ Although successive Mn oxidation-state changes have been proposed for birnessite-type oxides, the present CV curves do not uniquely identify each feature as a stoichiometric MnO_2_/MnOOH/Mn(OH)_2_ phase conversion. The peak labels, if retained in Fig. 7a, should therefore be treated as descriptive labels rather than proof of four individually resolved bulk reactions.

**Fig. 7:**
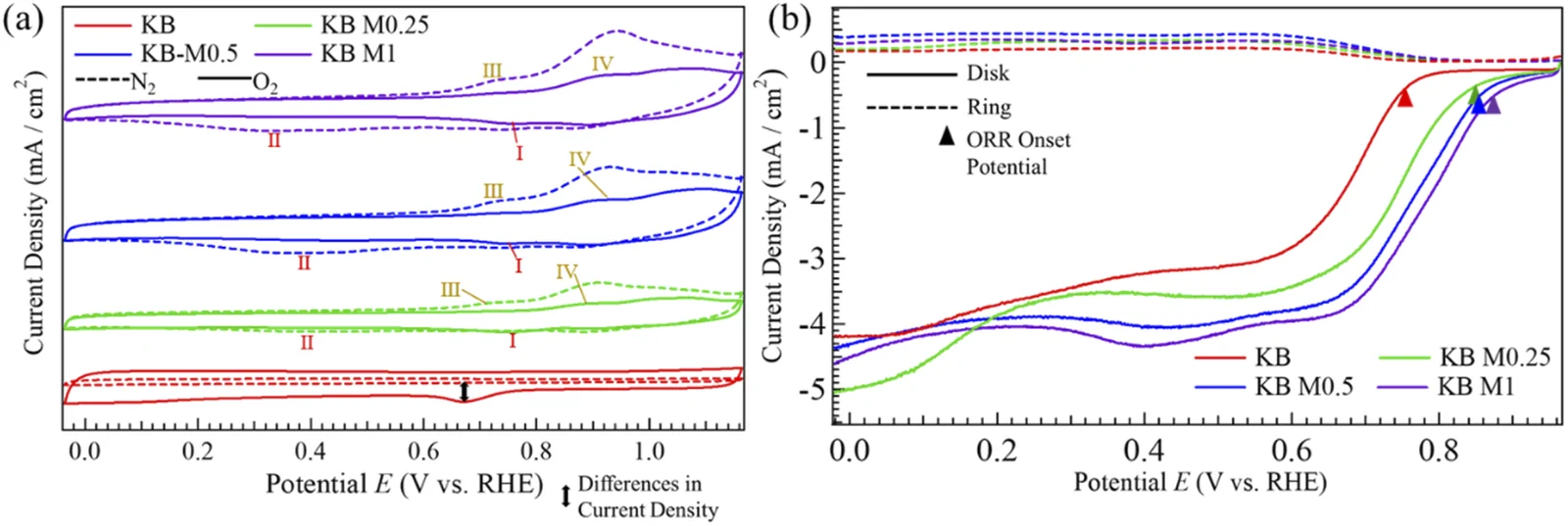
Electrochemical evaluation of KB, KB-M0.25, KB-M0.5, and KB-M1 in 0.1 M KOH: (a) CV curves recorded in N_2_- and O_2_-saturated electrolytes at 50 mV s^−1^, where the dashed and solid lines represent the responses measured under N_2_ and O_2_, respectively; and (b) background-corrected RRDE polarization curves recorded in O_2_-saturated electrolyte at 10 mV s^−1^ and 1500 rpm, where the solid and dashed lines represent the disk and ring responses, respectively. The ring potential was fixed at 0.5 V *vs.* RHE.

The larger CV response of the KB-M samples relative to KB is consistent with an added manganese-oxide-related contribution. Its increase with precursor concentration may reflect increasing oxide loading and pseudocapacitance, in agreement with the EDS trend in Table 1. Differences between the N_2_- and O_2_-saturated anodic curves further show that the overall electrode response depends on the gas environment. However, the present data do not establish that dissolved O_2_ chemically pre-oxidizes a quantified population of Mn^2+^ or Mn^3+^ sites before the anodic scan. Differences between the CNT- and KB-supported electrodes may arise from their combined differences in aggregate structure, surface area, surface chemistry, pretreatment, oxide deposition, and electrode transport; the CV peak separation is not used as a direct measurement of support conductivity.

The LSV results show that the onset potential increases from 0.758 V for KB to 0.849, 0.855, and 0.873 V *vs.* RHE for KB-M0.25, KB-M0.5, and KB-M1, respectively (Table 4). KB-M1 exhibits the highest measured onset potential among the composites examined in this study. The corresponding geometric current densities at 0.464 V *vs.* RHE are −3.65, −3.77, −3.91, and −4.21 mA cm^−2^, respectively. These trends are consistent with a greater manganese-oxide contribution as the precursor concentration increases, but they do not establish a monotonic relationship with the fitted Mn^3+^ fraction. In particular, the original fitted Mn^3+^ values vary non-monotonically, whereas the onset potential and geometric current magnitude increase across the KB-M series. The activity trend is therefore interpreted as the combined result of composition, oxide structure, electrode accessibility, and transport rather than as evidence for a single Mn-valence descriptor.^23,38^

**Table 4:** ORR onset potentials and RRDE parameters of KB, KB-M0.25, KB-M0.5, and KB-M1^a^

	ORR onset potential (V)	Electron transfer numbers	Peroxide yield, HO_2_^−^ (%)	Current density at (mA cm^−2^)
KB	0.758	3.06	46.8	−3.65
KB-M0.25	0.849	3.31	34.3	−3.77
KB-M0.5	0.855	3.16	42.2	−3.91
KB-M1	0.873	3.33	33.5	−4.21

a Electron-transfer number, HO_2_^−^ yield, and geometric current density were evaluated at 0.464 V *vs.* RHE. The onset potential is an independently determined characteristic.

The KB-series electron-transfer numbers and peroxide yields are shown in Fig. S10 and S11, respectively. At 0.464 V *vs.* RHE, the *n* values for KB, KB-M0.25, KB-M0.5, and KB-M1 are 3.06, 3.31, 3.16, and 3.33, respectively, while the corresponding HO_2_^−^ yields are 46.8%, 34.3%, 42.2%, and 33.5%. These paired values are internally consistent with eqn (1) and (2); small differences arising when the displayed *n* values are used to recalculate the peroxide yields result from numerical rounding. The selectivity varies non-monotonically with precursor concentration, indicating that increasing KMnO_4_ concentration does not produce a simple monotonic shift toward either the two- or four-electron reduction pathway. This trend may reflect combined effects of manganese-oxide loading, local surface structure, oxide aggregation, electrolyte accessibility, and electrode transport, and it is not attributed uniquely to the fitted Mn^3+^ fraction. The RRDE results describe the apparent net electron-transfer pathway under the stated measurement conditions but do not distinguish direct four-electron oxygen reduction from a sequential pathway involving initial HO_2_^−^ formation followed by its further reduction or decomposition.

KB-M1 shows a slightly larger geometric current magnitude than CNT-M1 at 0.464 V *vs.* RHE (−4.21 and −4.17 mA cm^−2^, respectively). SEM and EDS mapping show manganese-containing material distributed across the sampled KB aggregates (Fig. S3), while the capacitance-derived apparent area ratio is larger for the KB-M electrodes (Table S3). These observations are compatible with differences in oxide distribution and electrode accessibility. They do not demonstrate uniform deposition throughout the internal KB pore network, quantify accessible Mn sites, or establish that surface defects independently cause oxide deposition. Accordingly, KB-M1 is identified as the KB-series sample with the highest onset potential and geometric current magnitude, rather than as having a demonstrated superior intrinsic site activity.

#### 3.3.3 Capacitance-derived apparent area proxy

To compare the capacitance response of the CNT-M and KB-M composite electrodes, an apparent capacitance was obtained from CV measurements recorded at 0.01–0.05 V s^−1^ in the 0.1–0.3 V *vs.* RHE window in O_2_-saturated 0.1 M KOH. Because oxygen-reduction, manganese-oxide faradaic, and pseudocapacitive contributions may be present in this window, the measured slope is not assumed to represent a purely double-layer capacitance. A common reference capacitance, *C*_s_ = 0.04 mF cm^−2^, was used only to define a capacitance-derived apparent area ratio:

5 RF_app_ = *C*_app,geo_/*C*_s_

where *C*_app,geo_ is the apparent capacitance normalized to geometric electrode area. Accordingly, RF_app_ is dimensionless. An apparent area may be defined as *A*_app_ = RF_app_·*A*_geo_, but neither quantity is a direct measurement of the number or surface area of Mn catalytic sites. The detailed calculation is described in SI Section S1, and the corresponding CV curves are shown in Fig. S12 and S13 for the CNT-M and KB-M series, respectively.

The CNT-series apparent area ratio increases from 44.75 for CNT-M0.25 to 61.06 for CNT-M0.5 and then decreases to 50.67 for CNT-M1 (Table S3). The decrease from CNT-M0.5 to CNT-M1 is consistent with, but does not prove, particle coarsening or surface blocking. In contrast, the KB-series ratio increases from 197.57 for KB-M0.25 to 308.12 for KB-M0.5 and 359.95 for KB-M1. KB-M1 therefore has an apparent area ratio approximately 7.1 times that of CNT-M1 under the common reference-capacitance assumption. Because the supports and oxide loadings may have different specific and pseudocapacitive responses, this ratio is not interpreted as a 7.1-fold difference in accessible Mn area or active-site number.

Fig. S14 and S15 show the ORR currents normalized using the capacitance-derived apparent area ratio. For current expressed initially as geometric current density, the normalized quantity is

6 *j*_app_ = *j*_geo_/RF_app_

This normalization provides an empirical comparison of current relative to the capacitance-derived apparent area proxy. Because the capacitance may include support-dependent, pseudocapacitive, and oxygen-reduction contributions, the proxy is not a quantitative measurement of accessible Mn surface area. The normalized LSV response also contains mass-transport contributions and is therefore not treated as a purely kinetic current, turnover frequency, or intrinsic site activity. Higher normalized current does not by itself prove faster interfacial charge transfer. The results instead indicate differences in geometric ORR response and capacitance-derived electrode accessibility between the two support families.

Taken together, the capacitance-derived apparent area ratios and geometric ORR responses indicate support- and precursor-concentration-dependent differences in the electrochemical behavior of the composite electrodes. The larger apparent area ratios of the KB-M samples may be associated with the high reported specific surface area and aggregated porous structure of KB, as well as differences in surface chemistry, oxide loading, and electrode architecture. However, the present measurements do not quantify the accessible Mn surface area or independently establish uniform δ-MnO_2_ dispersion throughout the internal KB pore network. Likewise, the larger apparent area ratio-normalized currents of the CNT-M samples do not by themselves demonstrate superior intrinsic Mn-site activity or stronger electronic coupling at the oxide/carbon interface. The decrease in the apparent area ratio from CNT-M0.5 to CNT-M1 is consistent with the observed particle coarsening and possible surface blocking, although these mechanisms are not independently established. Overall, the results indicate that precursor concentration influences the balance among oxide loading, geometric ORR response, and capacitance-derived electrode accessibility, while the optimum condition depends on the performance metric considered.

#### 3.3.4 Long-term electrochemical stability

To assess the long-term electrochemical stability of the catalysts synthesized by SPP, CNT-M0.5 and KB-M1 were selected as representative samples from their respective series for chronoamperometric (CA) measurements. Specifically, KB-M1 was chosen as the representative of the KB series due to its highest ORR onset potential and largest magnitude of geometric current density at 0.464 V *vs.* RHE among the KB-M samples (Table 4). It also exhibited the highest capacitance-derived apparent area ratio in this series (Table S3). For the CNT series, although the apparent area ratio-normalized LSV curves show larger normalized current magnitudes for CNT-M1 and CNT-M0.25 over parts of the measured potential range (Fig. S14), this normalization does not establish higher intrinsic Mn-site activity. CNT-M0.5 was selected because it exhibited the highest ORR onset potential within the CNT-M series (Table 3), together with the largest capacitance-derived apparent area ratio in that series (Table S3). Both representative electrodes were subjected to CA measurements at 0.464 V *vs.* RHE and 1500 rpm for 20 000 s in O_2_-saturated 0.1 M KOH. The normalized current retention (*I*/*I*_0_) as a function of time is shown in Fig. 8, and the final retention values at *t* = 20 000 s are summarized in Table S4.

**Fig. 8:**
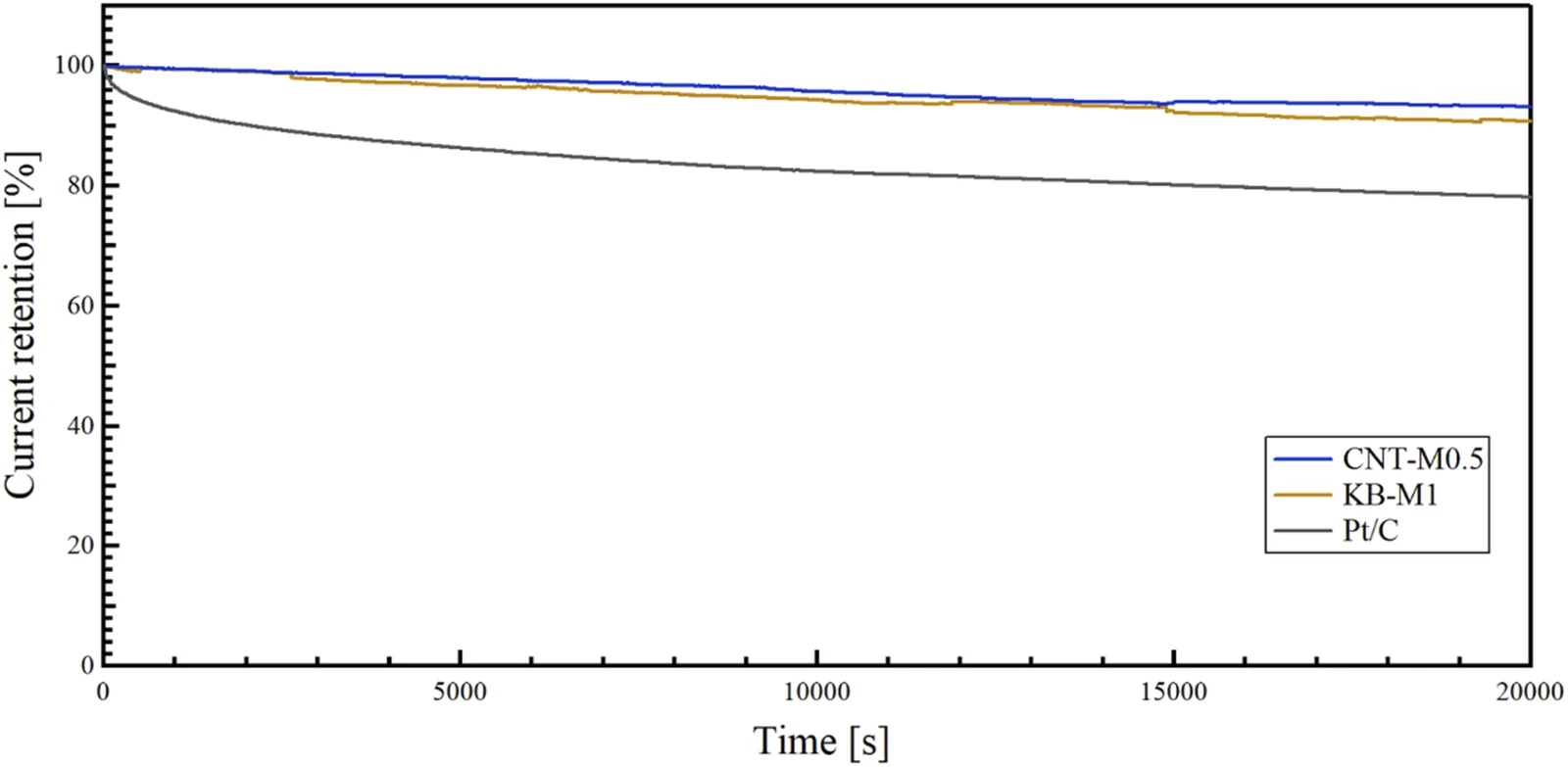
Current–time (*I*–*t*) chronoamperometric response (1500 rpm) of CNT-M0.5, KB-M1, and 20% Pt/C at 0.464 V in an O_2_-saturated 0.1 M KOH solution for 20 000 s.

As shown in Fig. 8, both CNT-M0.5 and KB-M1 exhibit an initial decrease in current followed by a comparatively stable response over the remainder of the measurement. In contrast, commercial Pt/C shows a more sustained decrease in current during the same test. The initial transient may involve equilibration of the catalyst layer and electrode–electrolyte interface, but its origin cannot be uniquely determined from the current–time curves. Quantitatively, the final current retentions of CNT-M0.5 and KB-M1 were 93.1% and 90.8%, respectively, compared with 78.1% for Pt/C under the same test conditions. The differences relative to Pt/C are 15.0 and 12.7 percentage points. CNT-M0.5 therefore showed the highest final current retention among the three electrodes evaluated in this test. Current retention demonstrates operational stability under these conditions, but does not uniquely identify a microscopic stabilization or degradation mechanism.

For context, pristine and Cu^2+^-modified birnessite have been reported to retain 87.4% and 94.8% of their initial currents, respectively, during a 10 000 s test at 0.4 V *vs.* RHE in O_2_-saturated 0.1 M KOH at 1500 rpm.^18^ In the present study, CNT-M0.5 and KB-M1 retained 93.1% and 90.8%, respectively, after 20 000 s at 0.464 V *vs.* RHE and 1500 rpm. A recent study of layered birnessite-based catalysts also reported potential-dependent current degradation.^46^ These comparisons indicate that stability depends on catalyst composition and measurement conditions. Because the test potentials and durations differ, the retention percentages are not used to establish a direct ranking among the catalysts.

Post-test Raman spectra, SEM images, and EDS maps are presented in Fig. S16 and S17, and the corresponding post-test EDS atomic compositions are summarized in Table S5. The samples were recovered following rinsing and drying, as described in Section 2.3. The characteristic carbon-support morphologies and detectable Mn and O signals remain after CA. The EDS values confirm the presence of Mn and O within the analyzed post-test regions but do not provide a quantitative measure of Mn retention or dissolution. Raman D/G features persist, while changes and apparent broadening occur in the oxide-related low-wavenumber region. These observations are compatible with retained manganese-containing material and possible evolution of its local environment. However, the post-test characterization examines the material remaining on the electrodes after testing, rinsing, and recovery, and possible changes introduced by these procedures must also be considered. The observations do not establish retention of an unchanged δ-MnO_2_ phase, Mn valence distribution, or atomic-scale interface bonding, and they do not quantify dissolution. Because post-test XRD and XPS measurements were not performed, changes in crystalline phase and surface Mn chemical states cannot be resolved from the present dataset.

The observed stability may involve the oxide response in alkaline electrolyte, support/electrode integrity, and interfacial contact, consistent with the broader relevance of oxide structure and carbon electrode chemistry.^18,26,42^ The persistence of the carbon morphology and Mn/O signals is consistent with retention of the oxide/carbon composite, but does not demonstrate complete suppression of oxide dissolution or detachment. The present data do not establish SPP-generated covalent Mn–C bonding as the primary stabilizing factor, nor do they identify Pt/C current loss uniquely with ripening, dissolution, or carbon corrosion. Overall, CNT-M0.5 and KB-M1 exhibit higher current retention than commercial Pt/C during the specified 20 000 s CA test, while the microscopic origins of their stability remain to be established.

### 3.4 Comparison with previously reported MnO_2_-based ORR catalysts

To contextualize the electrochemical performance and preparation conditions of the catalysts synthesized in this work, Table 5 summarizes selected literature data together with the preparation and electrochemical measurement conditions reported in the original studies. The comparison includes the catalyst composition, principal preparation conditions, electrolyte, rotation rate, catalyst loading where available, onset and half-wave potentials, and current density at the potential reported in each study. The current densities are presented at the potentials at which they were reported rather than being converted or read from polarization curves at a common potential. Because catalyst composition, loading, rotation rate, onset-potential definition, current-density evaluation potential, and synthesis boundaries differ among the studies, the comparison is contextual and is not used to establish a universal performance ranking.

**Table 5:** Comparison of the preparation and reported alkaline ORR performance of the present composites with selected manganese-oxide-based catalysts^d^

Catalyst	Preparation conditions^a^	Electrolyte	Rotation rate/rpm	Catalyst loading/mg cm^−2^	Onset potential/V *vs.* RHE	Half-wave potential/V *vs.* RHE	Reported current-density metric^b^	Ref.
KB-M1	SPP, 10 min, atmospheric pressure, without external heating	0.1 M KOH	1500	0.298^c^	0.873	NR	−4.21 mA cm^−2^ at 0.464 V	This work
CNT-M0.5	SPP, 10 min, atmospheric pressure, without external heating	0.1 M KOH	1500	0.298^c^	0.804	NR	−3.75 mA cm^−2^ at 0.464 V	This work
CNT-graphene-α-MnO_2_ hybrid	CNT acid pretreatment at 130 °C for 12 h, followed by hydrothermal treatment at 180 °C for 8 h	O_2_-saturated 0.1 M KOH	1600	0.72	0.94	0.855	NR	7
Birnessite	Mixing for 2 h, stirring for 30 min after KOH addition, and aging at 60 °C for 16 h	O_2_-saturated 0.1 M KOH	1500	NR	0.80	0.64	4.6 mA cm^−2^ in magnitude at 0.00 V	18
Cu^2+^/birnessite	Mixing for 2 h, stirring for 30 min after KOH addition, and aging at 60 °C for 48 h	O_2_-saturated 0.1 M KOH	1500	NR	0.87	0.67	4.6 mA cm^−2^ in magnitude at 0.00 V	18
50 wt% MnO_2_/N-doped CNT	Improved impregnation of functionalized N-doped CNTs; nominal MnO_2_ content of 50 wt%	Alkaline electrolyte; concentration NR	NR	NR	0.98	0.84	−2.1 mA cm^−2^ at *E*_1/2_ = 0.84 V	27
MnO_2_/GO	Hydrothermal preparation of MnO_2_ nanorods at 140 °C for 12 h, followed by composite formation with GO at 90 °C for 30 min	O_2_-saturated 0.1 M KOH	1600	0.30	0.93	0.78	5.10 mA cm^−2^ in magnitude, reported as the limiting current density	47

a NR, not reported.

b The listed time denotes the principal reaction or aging step and does not necessarily include pretreatment, washing, recovery, or drying.

c Current densities are presented at the potentials or electrochemical regions reported in the original studies without conversion or interpolation. Values obtained at different potentials or using different current-density metrics are not directly comparable. The MnO_2_/GO value is the reported limiting-current magnitude.

d The loading of the present catalysts was calculated from 7.5 µL of a 5 mg mL^−1^ ink deposited on a 0.126 cm^2^ disk.

Within the present series, KB-M1 exhibits the highest onset potential (0.873 V *vs.* RHE) and the largest magnitude of geometric current density at 0.464 V (−4.21 mA cm^−2^). The CNT-graphene-α-MnO_2_ hybrid reported by Chen *et al.* exhibits a more positive onset potential of 0.94 V *vs.* RHE under its reported conditions,^7^ while pristine and Cu^2+^-modified birnessites reported by Ochirkhuyag *et al.* show onset potentials of 0.80 and 0.87 V *vs.* RHE, respectively.^18^ These values provide context for the present results but are not directly ranked because the catalyst compositions, loadings, rotation rates, onset-potential definitions, and reported current-density potentials differ.

The composite-formation step used in the present work consists of a 10 min SPP treatment at atmospheric pressure without external heating. In comparison, Chen *et al.* employed an 8 h hydrothermal treatment at 180 °C after a separate 12 h nitric-acid pretreatment of the CNT support, while Ochirkhuyag *et al.* used aging steps of 16 h for pristine birnessite and 48 h for Cu^2+^-modified birnessite at 60 °C.^7,18^ This comparison concerns the principal reaction or aging step and does not represent the total preparation time, which may also include pretreatment, mixing, washing, recovery, and drying. Electrical energy consumption was not measured in the present study; therefore, the shorter SPP step is not used to claim a quantified energy-efficiency advantage. Nevertheless, the 10 min treatment demonstrates that ORR-active manganese-oxide/carbon composites can be obtained using a substantially shorter principal composite-formation step than the selected hydrothermal and aging routes.

The 50 wt% MnO_2_/N-doped CNT catalyst reported by Andersen *et al.* exhibited an onset potential of 0.98 V and a half-wave potential of 0.84 V *vs.* RHE, whereas the MnO_2_/GO composite reported by Ahmed *et al.* exhibited corresponding values of 0.93 and 0.78 V *vs.* RHE.^27,47^ These values were obtained under measurement and loading conditions different from those used in the present study and are therefore included for contextual comparison rather than direct performance ranking.

## 4 Conclusions

In this study, carbon-supported birnessite-type manganese-oxide composites were prepared by the solution plasma process (SPP) using a 10 min discharge step at atmospheric pressure without external heating. CNT and KB supports were investigated at three KMnO_4_ precursor concentrations. XRD, SEM, and EDS results were consistent with the formation and precursor-concentration-dependent deposition of poorly crystalline birnessite-type manganese oxide on both supports. Greater Mn atomic percentages were detected in the KB-based composites, whereas the CNT- and KB-based samples exhibited different oxide-deposition and aggregation characteristics. Because the supports also differ substantially in surface area, surface chemistry, porosity, graphitic organization, and pretreatment, these results demonstrate support-dependent composite formation rather than an independently isolated carbon-morphology effect.

XPS revealed support- and precursor-concentration-dependent differences in the Mn, O, and C spectral responses. Oxygen-containing carbon species were already present in the pre-synthesis supports, and the low-binding-energy C 1s contribution near 282.0–282.5 eV remained of uncertain origin. The spectra therefore do not establish SPP-induced carbon functionalization, direct Mn–C bonding, a unique interfacial charge-transfer pathway, or oxygen-vacancy concentrations. The fitted Mn^3+^ fractions increased across the CNT-M series and varied non-monotonically across the KB-M series, whereas the electrochemical trends followed different concentration dependences. Thus, the alkaline ORR response cannot be explained by a single fitted Mn^3+^ fraction and likely reflects the combined effects of oxide formation, surface chemistry, electrode accessibility, and transport.

In O_2_-saturated 0.1 M KOH, CNT-M0.5 exhibited the highest onset potential within the CNT-M series (0.804 V *vs.* RHE), while KB-M1 exhibited the highest onset potential among the synthesized composites (0.873 V *vs.* RHE) and a geometric current density of −4.21 mA cm^−2^ at 0.464 V. CNT-M1 reached an apparent electron-transfer number of 3.94 under the evaluated RRDE conditions. After 20 000 s of chronoamperometry at 0.464 V *vs.* RHE and 1500 rpm, CNT-M0.5 and KB-M1 retained 93.1% and 90.8% of their initial currents, respectively, compared with 78.1% for commercial Pt/C under the same conditions. Post-test Raman, SEM, and EDS measurements were consistent with the continued presence of manganese-containing material but did not establish an unchanged oxide phase, Mn valence distribution, or atomic-scale interface.

Overall, the results demonstrate a short SPP route to ORR-active manganese-oxide/carbon composites and identify support selection and precursor concentration as coupled variables affecting composite formation and alkaline ORR performance. Further work using matched supports, quantitative Mn loading, interface-sensitive characterization, and practical zinc–air-cell testing is required to resolve the underlying mechanisms and device-level relevance.

## Author contributions

Conceptualization and methodology, T. Ishizaki.; experimental and data analysis, K. Y., K. O., T. S., T. Iwato., and G. P.; writing – original draft preparation, K. Y. and T. Ishizaki; writing – review and editing, T. Ishizaki.; supervision, T. Ishizaki.; project administration and funding acquisition, T. Ishizaki. All authors have read and agreed to the published version of the manuscript.

## Conflicts of interest

There are no conflicts to declare.

## Supplementary Material

RA-016-D6RA06750F-s001

## Data Availability

The data supporting the findings of this study are included in the article and its supplementary information (SI). Supplementary information: additional structural, spectroscopic, microscopic, and electrochemical data, together with the XPS fitting parameters, capacitance-derived apparent area analysis, RRDE results, and post-chronoamperometry characterization. See DOI: https://doi.org/10.1039/d6ra06750f.
