# Supplementary material for: Solution plasma synthesis of birnessite-type MnO2/carbon composites: support-dependent surface chemistry and alkaline oxygen reduction performance
Source: RSC Adv. 2026 Nov 2;16(49):67683–700. doi: 10.1039/d6ra06750f (PMC13725866; doi:10.1039/d6ra06750f)

Supplementary Information (SI) for RSC Advances.
This journal is © The Royal Society of Chemistry

Electronic Supplementary Information

Solution Plasma Synthesis of Birnessite-Type MnO_2/Carbon Composites: Support-Dependent Surface Chemistry and Alkaline Oxygen Reduction Performance

Kouki Yamamoto[1], Kentaro Ogihara[1], Taiyo Suzuki[1], Toshiki Iwato[1],
Gasidit Panomsuwan[2], Takahiro Ishizaki[3*]

[1]Materials Science and Engineering, Graduate School of Engineering and Science, Shibaura Institute of Technology, Toyosu, Koto-Ku, Tokyo, 135-8548, Japan

[2]Department of Materials Engineering, Faculty of Engineering, Kasetsart University, Bangkok 10900, Thailand

[3]College of Engineering, Shibaura Institute of Technology, Toyosu, Koto-Ku, Tokyo, 135-8548, Japan

*Corresponding author: ishizaki@shibaura-it.ac.jp

S1. Determination of Apparent Capacitance and Capacitance-Derived Area Ratio for Current Normalization

The slope of Δj/2 versus scan rate v gives an apparent geometric capacitance $C_{\mathrm{app,geo}}$ when j is normalized by the geometric disk area. Δ$j = j_{\mathrm{a}} - j_{\mathrm{c}}$ is evaluated using a consistent sign convention, and v is expressed in V s⁻^{1} when the resulting capacitance is reported in mF cm⁻^{2}. Using a common reference $C_{\mathrm{s}} = 0.04$ mF cm^{-2}, $RF_{\mathrm{app}} = C_{\mathrm{app,geo}}/C_{\mathrm{s}}$ is dimensionless. The corresponding apparent area is $A_{\mathrm{app}} = RF_{\mathrm{app}} \times A_{\mathrm{geo}}$, with $A_{\mathrm{geo}} = 0.126$ cm^{2}. If capacitance is instead obtained from total current I, the total capacitance C_{app} has units of mF and $A_{\mathrm{app}} = C_{\mathrm{app}}/C_{\mathrm{s}}$.

The apparent area-normalized current is $j_{\mathrm{app}} = I/A_{\mathrm{app}} = j_{\mathrm{geo}}/RF_{\mathrm{app}}$. Geometric current density is not divided by an area in cm^{2}. The analysis assumes a common reference capacitance and may include oxide pseudocapacitance or ORR contributions; it therefore provides an electrode-area proxy rather than a uniquely determined Mn ECSA, turnover frequency, or mass-transport-corrected kinetic current.

Table S1: Representative binding energies and ranges of the XPS fitting parameters obtained for the six composite samples.

Core level	Fitted components	Representative binding energy / eV	Observed FWHM range / eV	Observed Lorentzian/Gaussian mixing-parameter range
Mn $2p$	Mn^{3+} $2p_{3/2}$, Mn^{4+} $2p_{3/2}$, Mn^{3+} $2p_{1/2}$, and Mn^{4+} $2p_{1/2}$	642.1, 643.1, 653.6, and 654.6, respectively	1.8–2.6, 2.0–3.3, 2.0–2.9, and 2.4–4.8, respectively	30:70
O 1s	Lattice oxygen, hydroxyl-related oxygen, and carbonyl-related oxygen	530.1, 531.5, and 535.2, respectively	0.9-1.1, 1.5–2.6, and 1.8–2.1, respectively	30:70
C 1s	Low-binding-energy, main C–C/C=C, and carbonyl-related contributions	281.8, 284.6, and 288.5, respectively	0.8–1.4, 1.6–2.0, and 1.9–2.3, respectively	30:70

Binding energies were charge-corrected using the main C 1s contribution at 284.6 eV. A Shirley background and mixed Lorentzian–Gaussian line shapes were used. The same number of components and component assignments were applied to the corresponding core-level spectra. The reported FWHM and Lorentzian/Gaussian mixing-parameter ranges represent the minimum and maximum fitted values obtained across the six composite samples.

Table S2: Relative fitted component areas (%) of the C 1s spectra shown in Fig. S4. The low-binding-energy contribution is of uncertain origin and is not assigned uniquely to Mn–C or Mn–O–C bonding. The reported areas are model-dependent spectral quantities rather than interfacial-bond concentrations.

	Low-binding-energy contribution	Main carbon contribution (C–C/C=C)	Carbonyl-related contribution (C=O)
CNT-M0.25	19.2	73.3	7.41
CNT-M0.5	21.6	74.7	3.58
CNT-M1	3.93	90.4	5.58
KB-M0.25	16.8	77	6.09
KB-M0.5	2.21	91.3	6.39
KB-M1	1.77	91.2	6.95

Table S3 Geometrically normalized apparent capacitance ($C_{app,geo}$) and capacitance-derived apparent area ratio for CNT-M0.25, CNT-M0.5, CNT-M1, KB-M0.25, KB-M0.5, and KB-M1. The dimensionless apparent area ratio was calculated as ($C_{app,geo}/C_s$), using a reference capacitance (C_s) of 0.04 mF cm^{-2}.

	$C_{app,geo}$ [mF/cm^2]	Capacitance-derived apparent area ratio [dimensionless]
(a) CNT-M0.25	1.79	44.75
(b) CNT-M0.5	2.44	61.06
(c) CNT-M1	2.03	50.67
(d) KB-M0.25	7.90	197.57
(e) KB-M0.5	12.32	308.12
(f) KB-M1	14.40	359.95

*The apparent area ratio was calculated by dividing the geometrically normalized apparent capacitance by a reference capacitance of 0.04 mF cm^{-2}. This dimensionless ratio is used as a comparative capacitance-derived proxy and is not interpreted as a directly measured ECSA.

Table S4 Current retention of CNT-M0.5, KB-M1, and commercial Pt/C after 20,000 s of chronoamperometric operation at 0.464 V vs RHE and 1500 rpm in O_2-saturated 0.1 M KOH.

	I/I_0 (t = 20000) [%]
CNT-M0.5	93.1
KB-M1	90.8
Pt/C	78.1

Table S5 EDS atomic compositions measured for CNT-M0.5 and KB-M1 recovered after the chronoamperometry test.

Sample	C / at.%	O / at.%	Mn / at.%
CNT-M0.5 after CA	98.6	1.31	0.08
KB-M1 after CA	94.3	3.19	2.48

*The values represent the atomic compositions of the analyzed regions of the recovered post-CA samples. Because EDS provides local, normalized elemental compositions and the samples underwent rinsing, drying,

and recovery before analysis, these values should not be interpreted as quantitative Mn retention or dissolution measurements.

Figure S1: TEM images of the carbon supports before composite synthesis: (a,b) acid-treated CNT and (c,d) pristine KB.

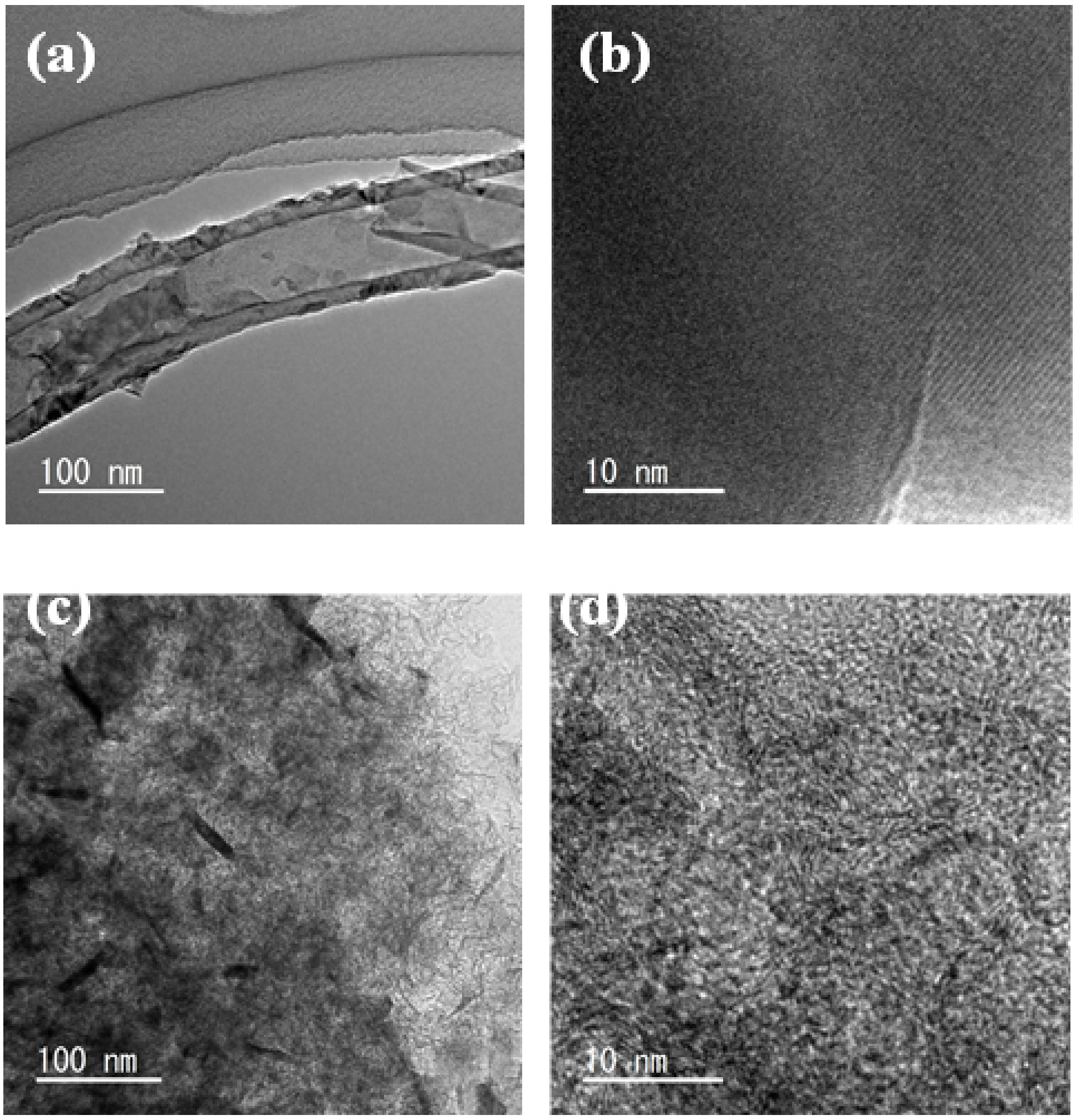

Figure S2: Elemental Mn, O, and C mapping images of (a) CNT-M0.5 and (b) CNT-M1.

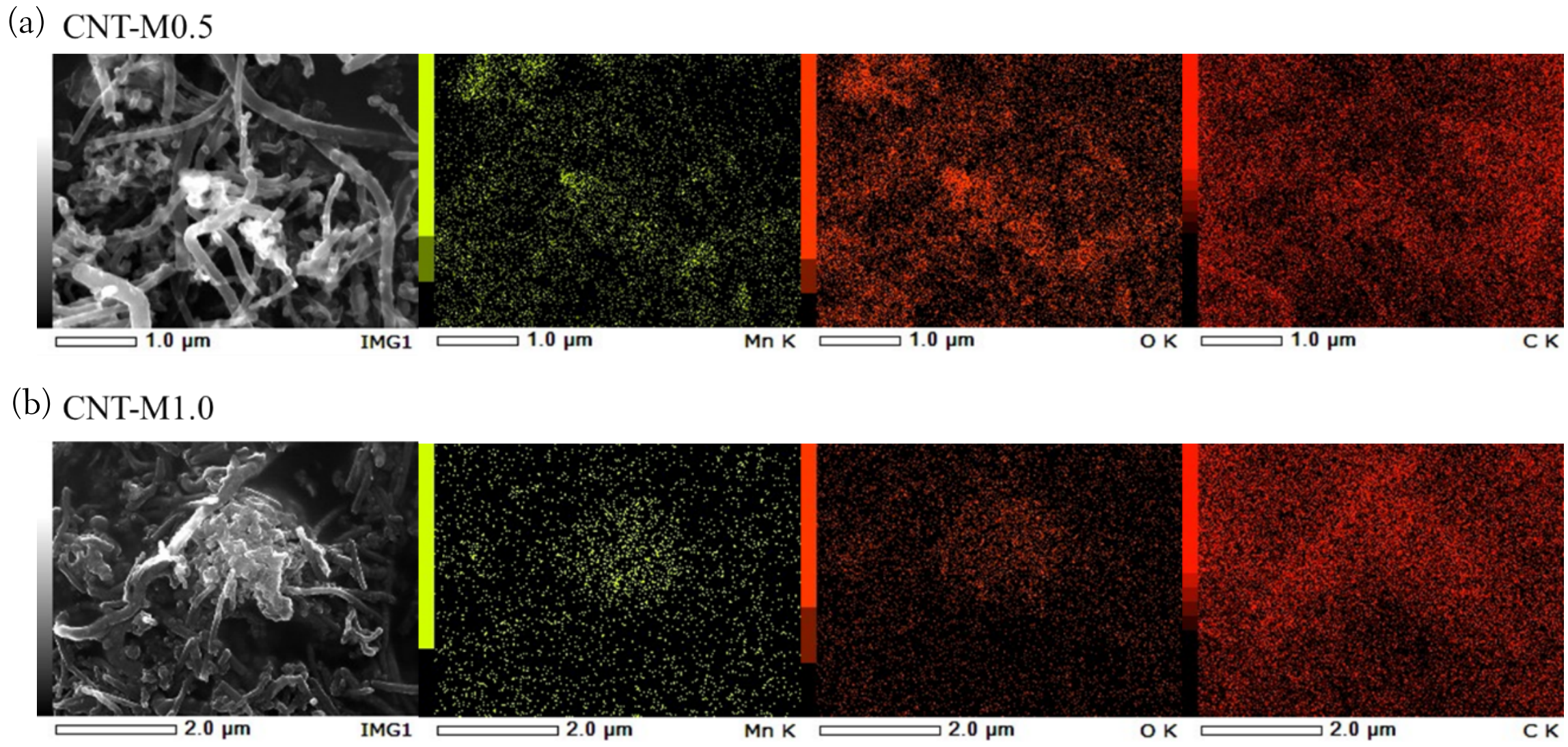

Figure S3: Elemental Mn, O, and C mapping images of (a) KB-M0.5 and (b) KB-M1.

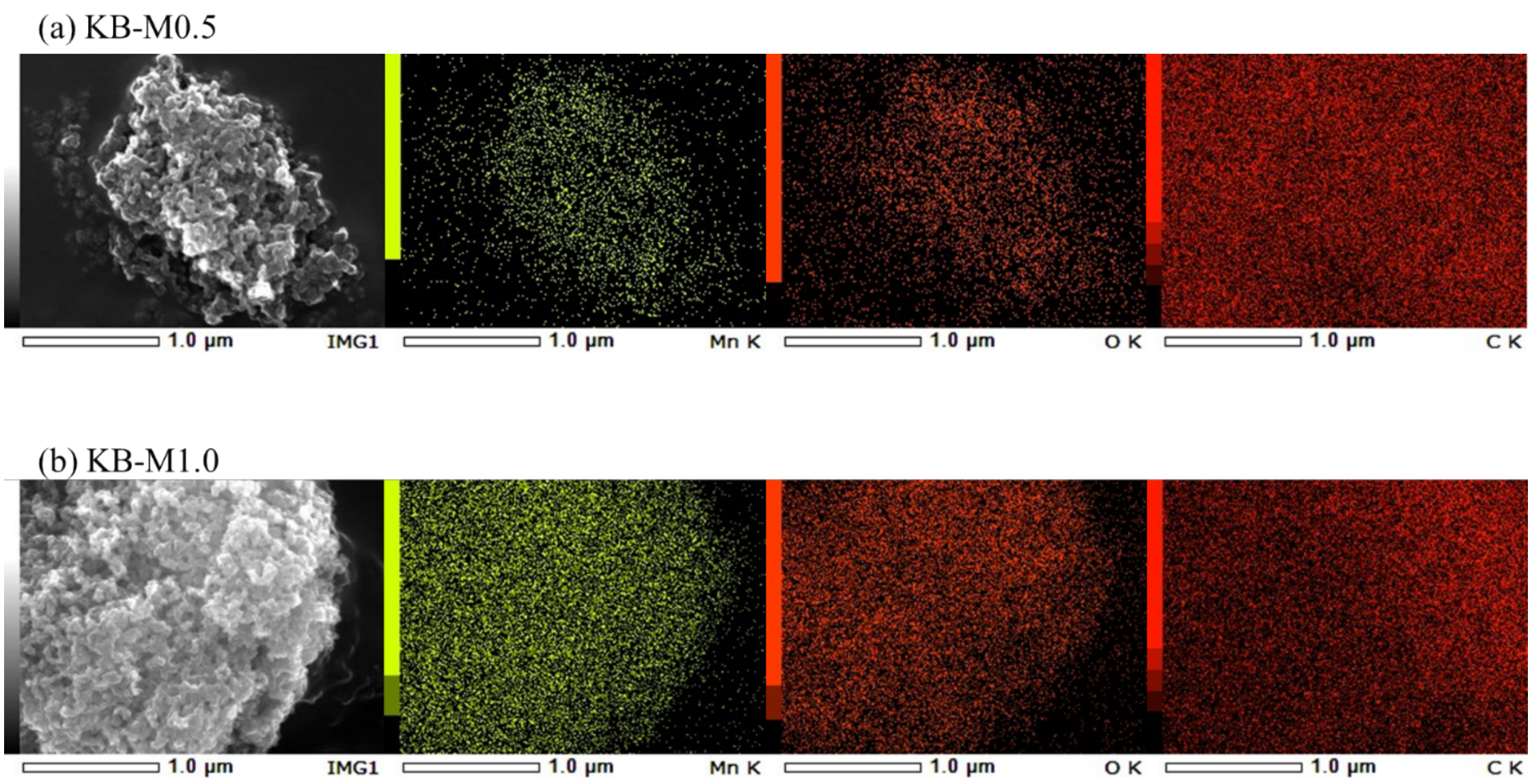

Figure S4: XPS C 1*s* spectra of (a) CNT-M0.25, (b) CNT-M0.5, (c) CNT-M1, (d) KB-M0.25, (e) KB-M0.5, and (f) KB-M1. The low-binding-energy contribution is of uncertain origin and is not assigned to Mn–C bonding.

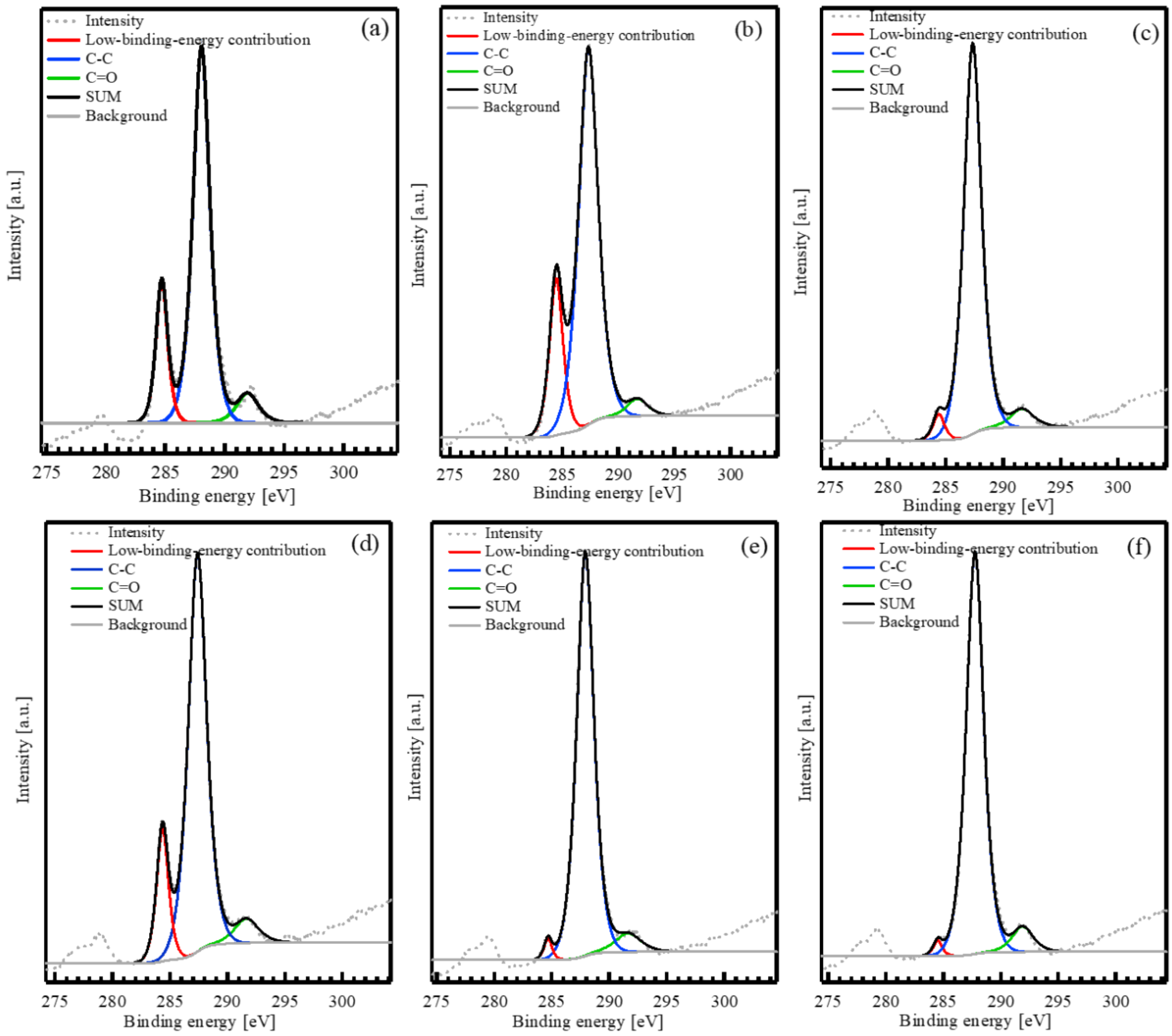

Figure S5: XPS O 1*s* spectra of the (a) acid-treated CNT and (b) KB supports before composite synthesis.

(a)

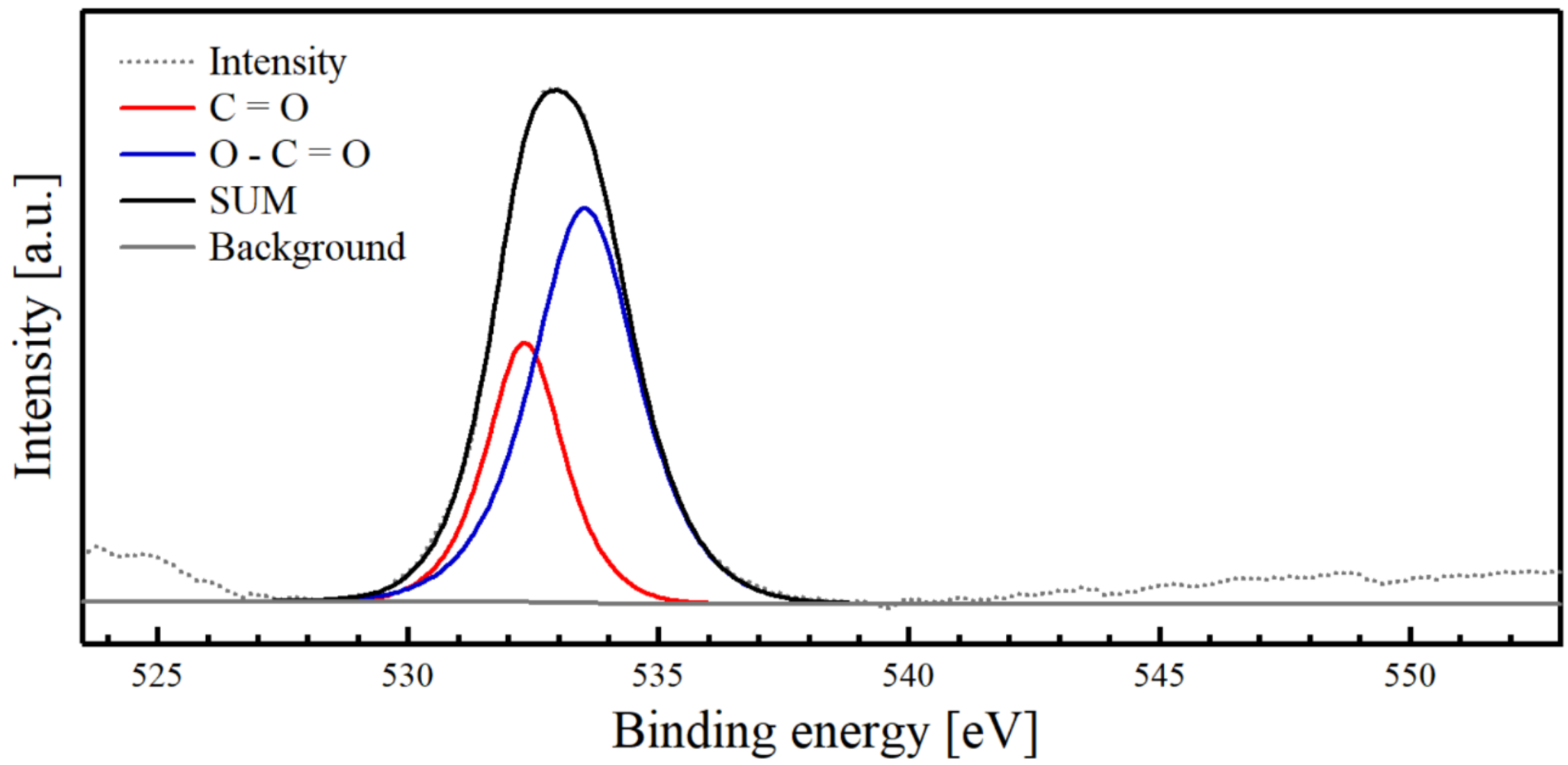

(b)

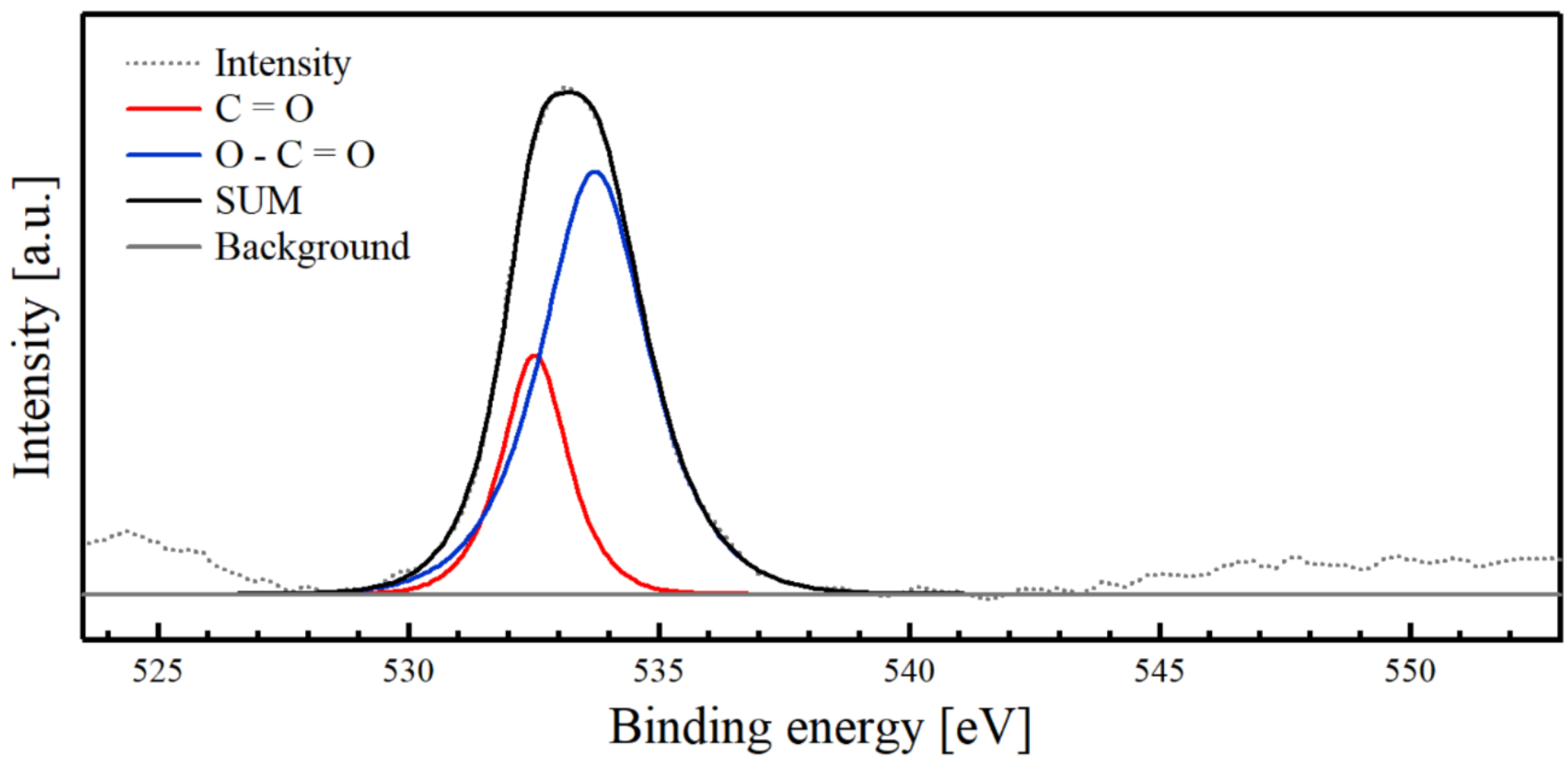

Figure S6: XPS C 1*s* spectra of (a) the acid-treated CNT and (b) the KB support before composite synthesis..

(a)

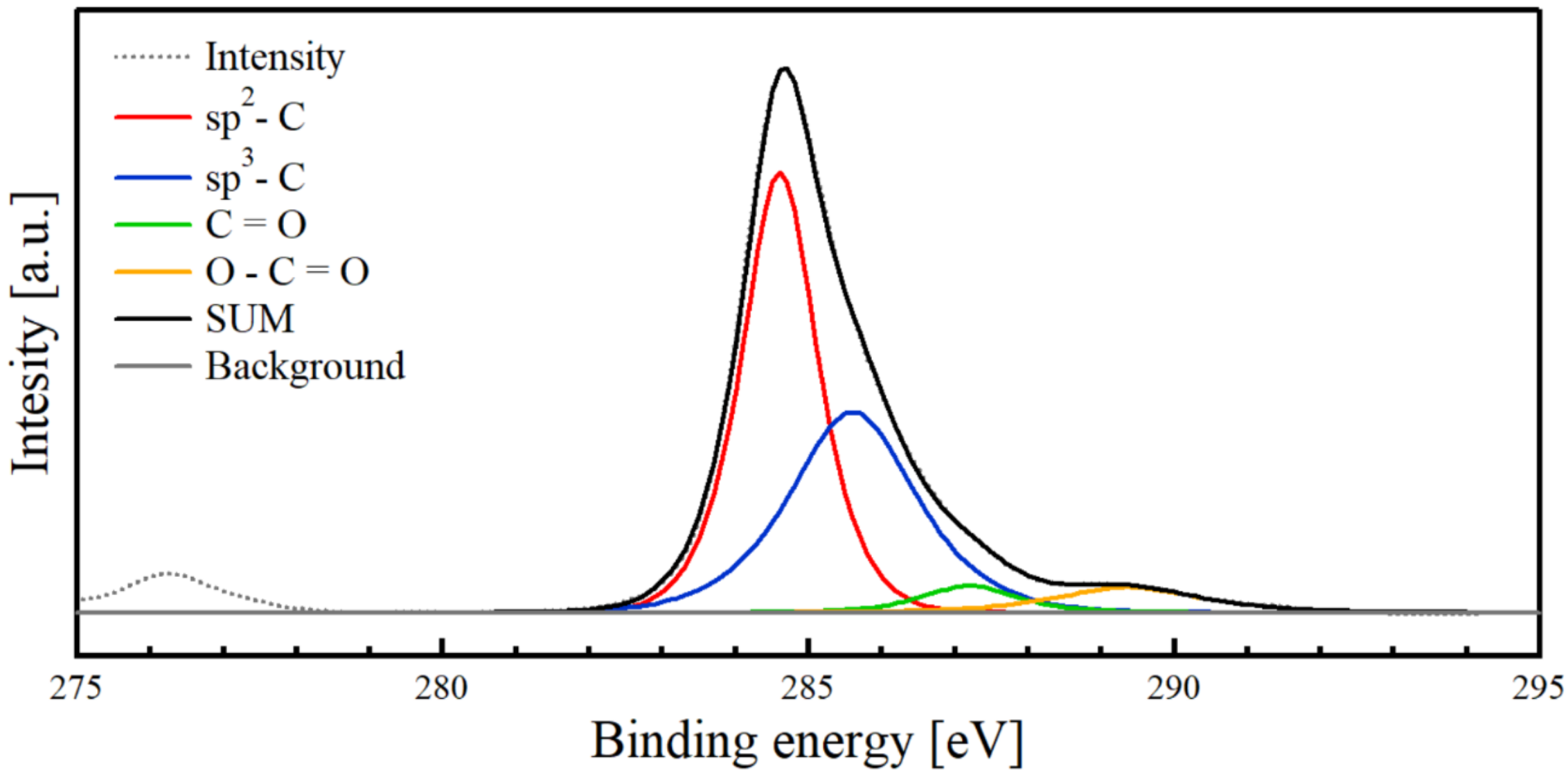

(b)

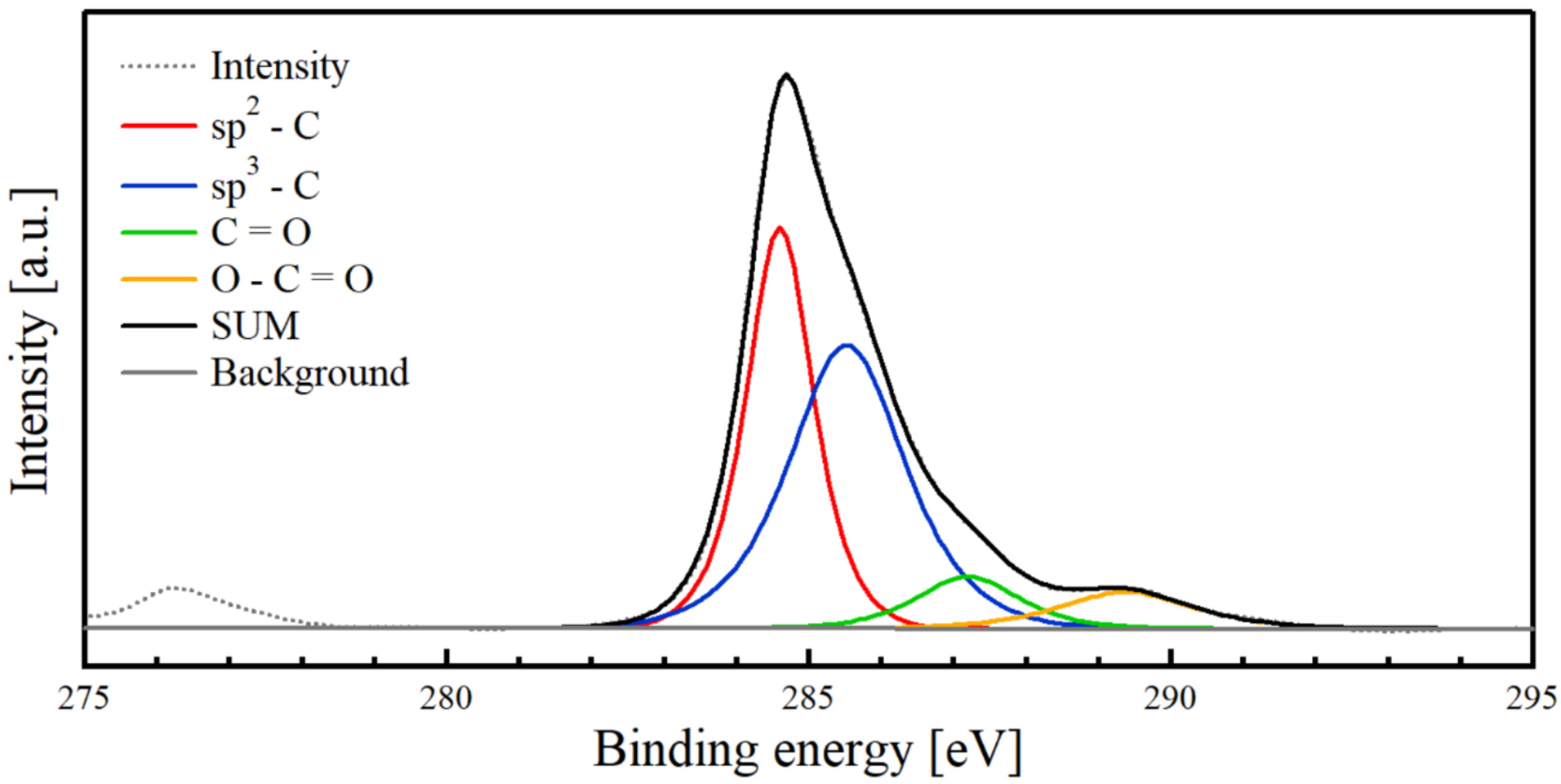

Figure S7: Raman spectra of (a) acid-treated CNT and CNT-M0.5 and (b) pristine KB and KB-M1, acquired using 532 nm excitation at a laser power of 0.4 mW

(a)

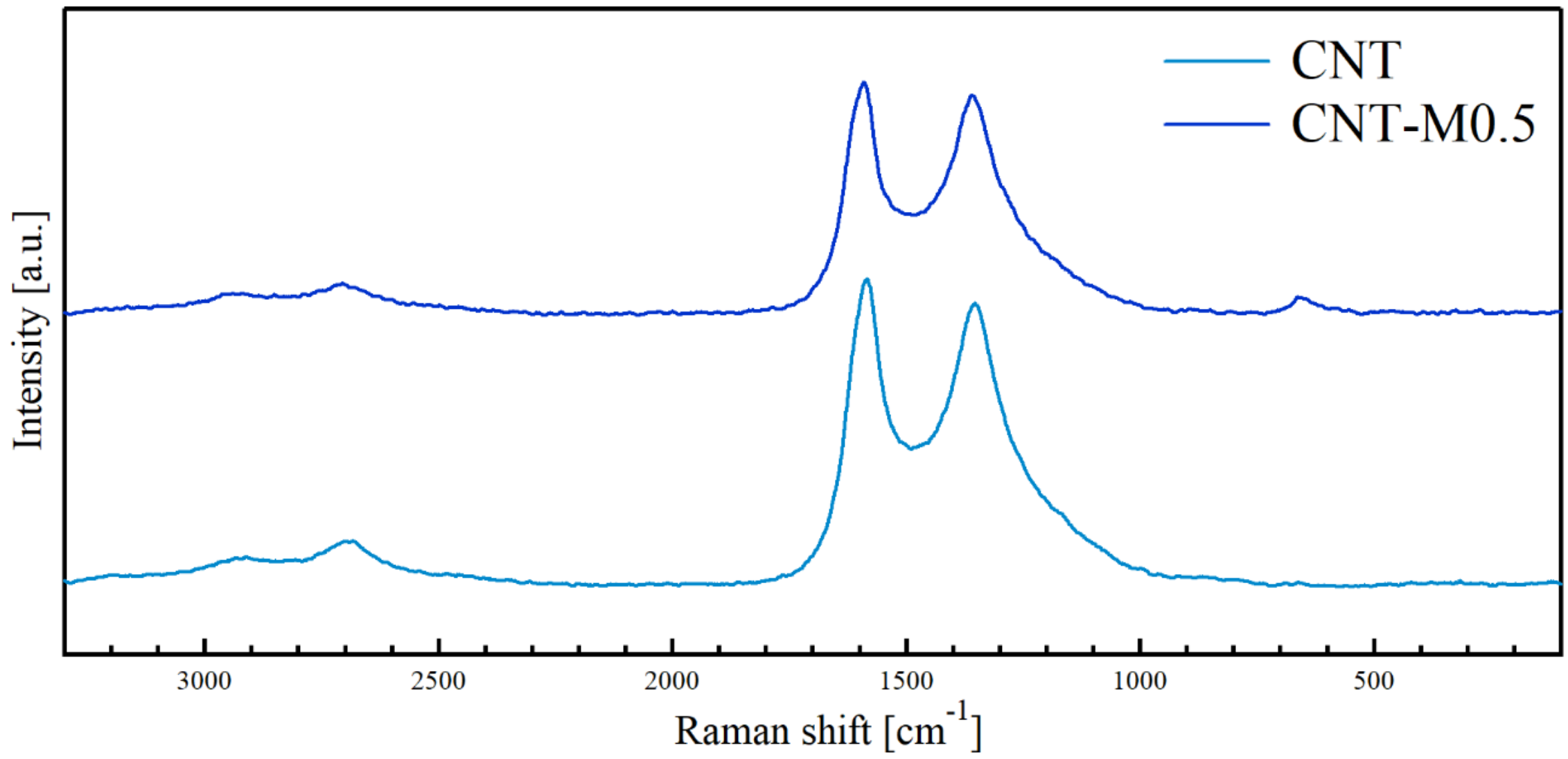

(b)

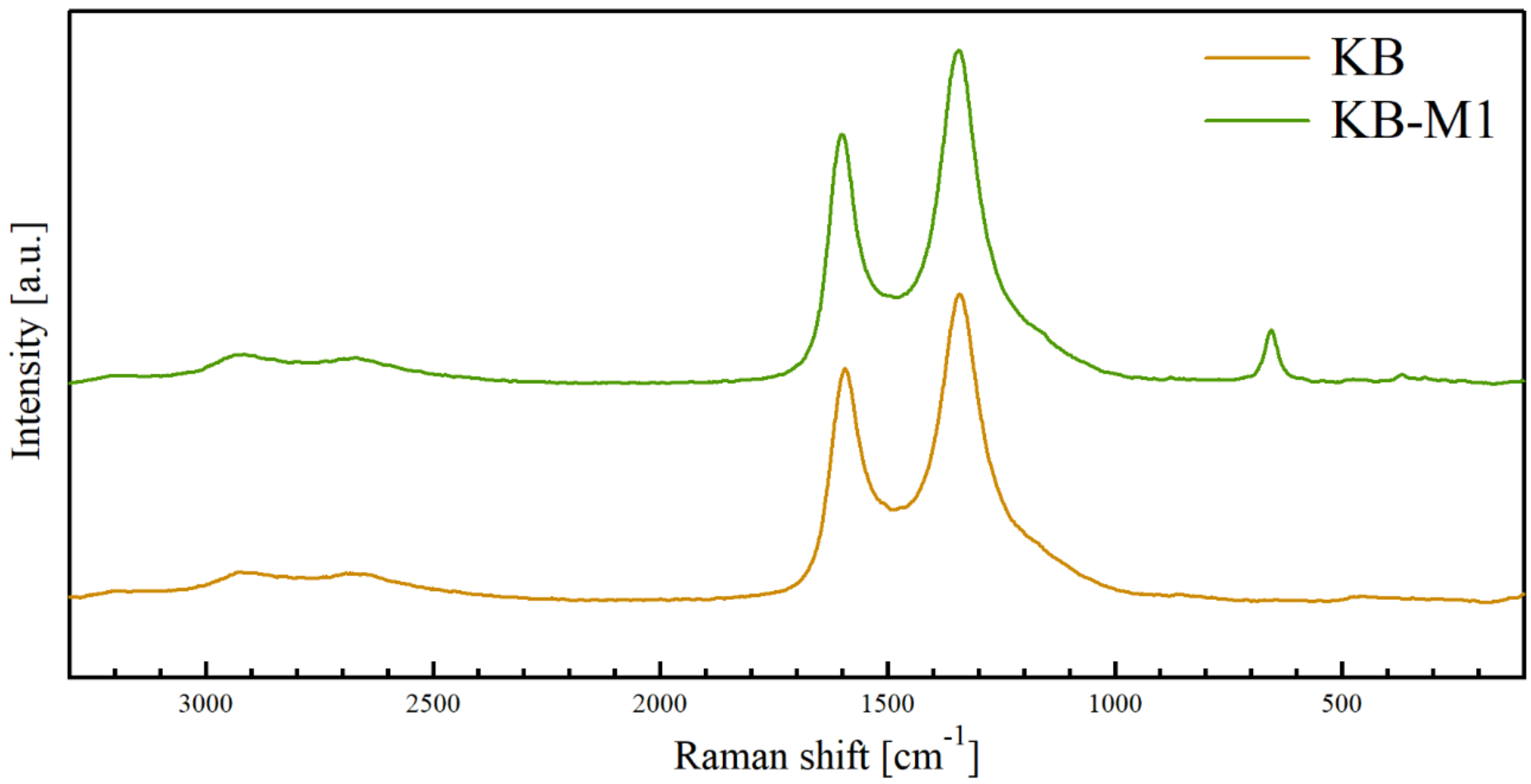

Figure S8: W 4*f* regions of CNT-M0.5 and KB-M1. No quantitative upper concentration limit is inferred without a validated detection-limit analysis.

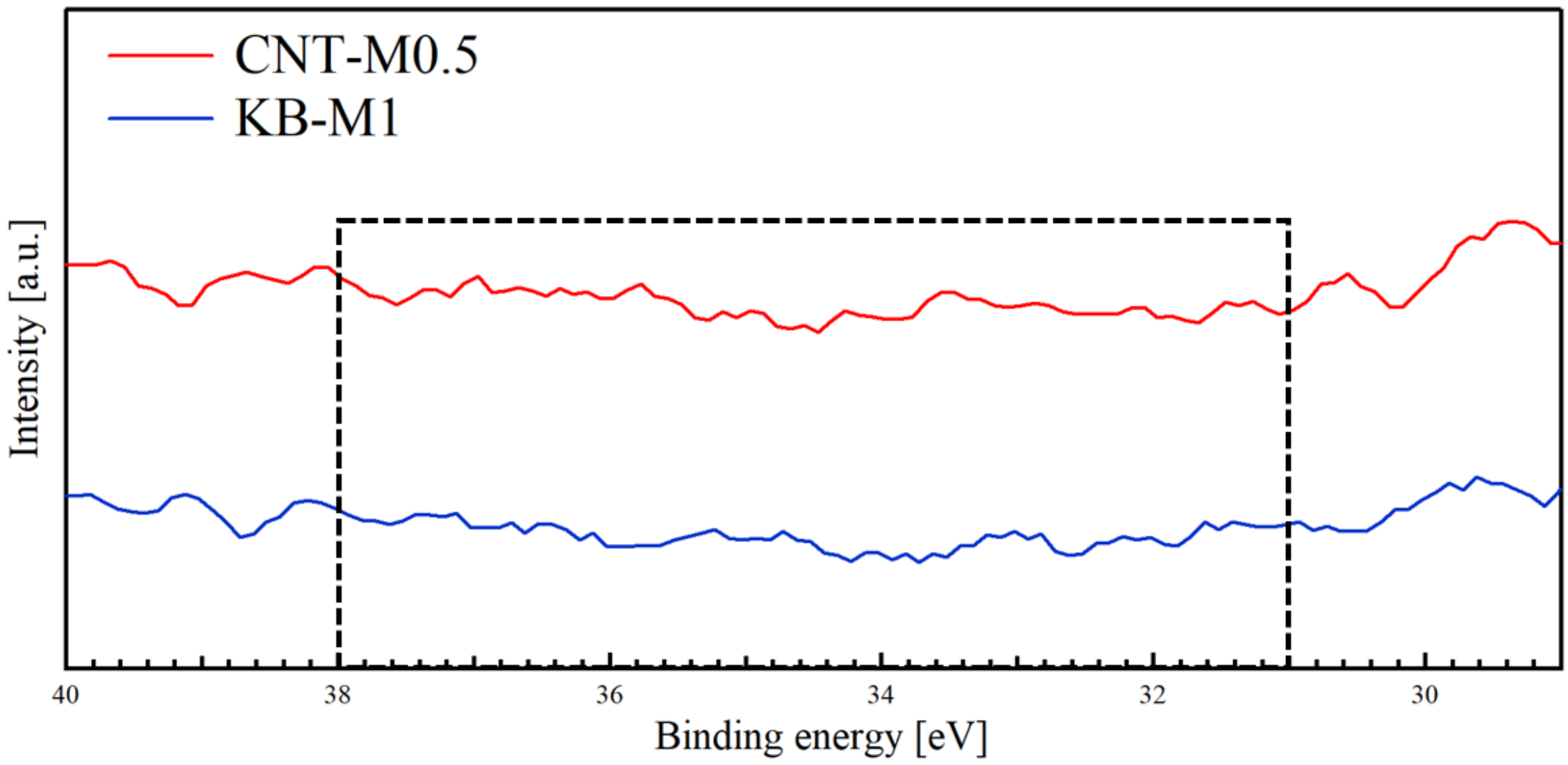

Figure S9: Background-corrected RRDE polarization curves for (a) the CNT-M series and (b) the KB-M series in O_2-saturated 0.1 M KOH at 10 mV s^{-1} and 1500 rpm, together with the response of commercial 20 wt% Pt/C measured under the same conditions. The solid and dashed lines represent the disk and ring responses, respectively. Background correction was performed by subtracting the corresponding current measured in N_2-saturated electrolyte. The ring potential was fixed at 0.5 V vs RHE.

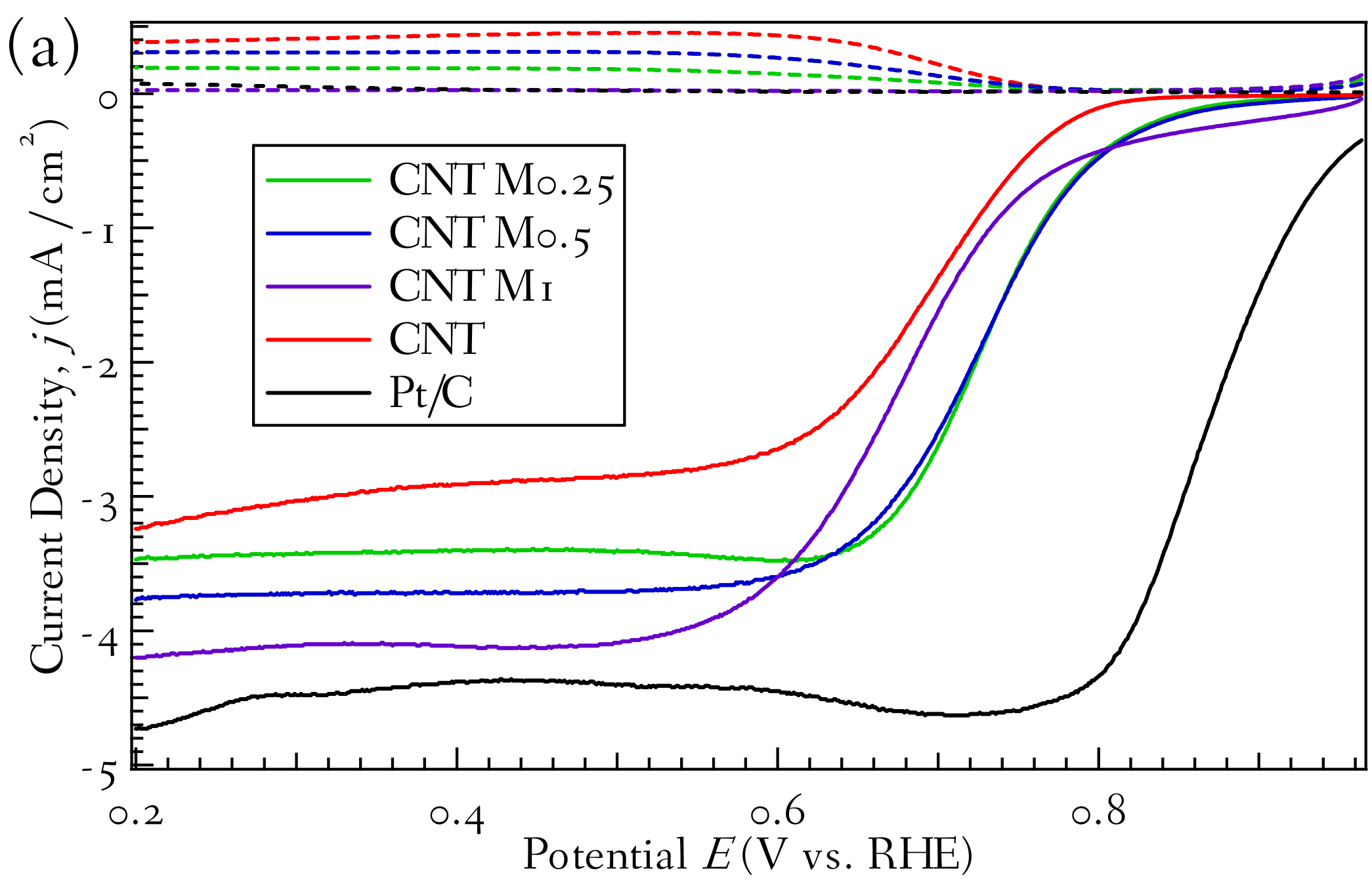

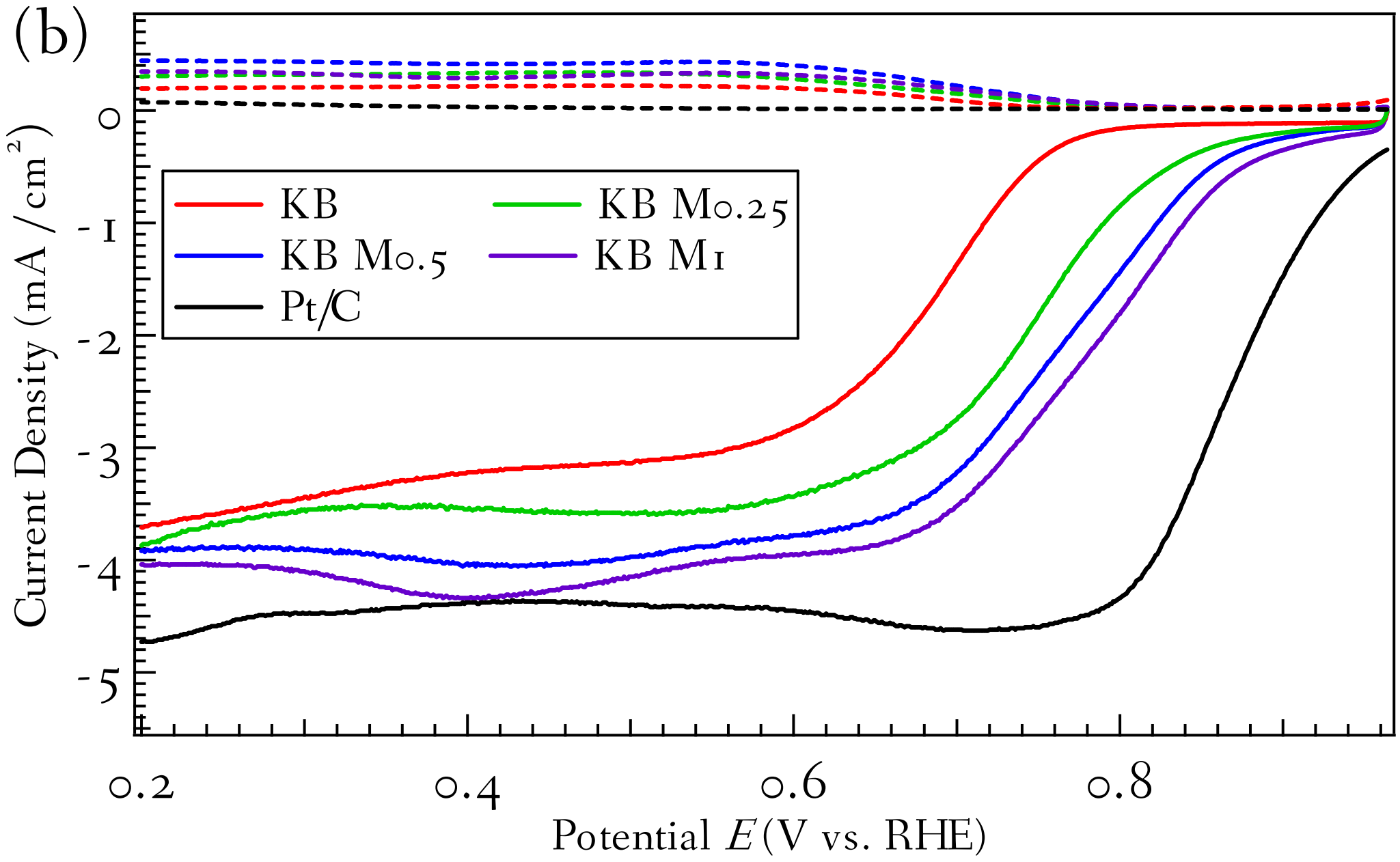

Figure S10: Change in electron transfer number (n) derived from rotating ring-disk electrode (RRDE) measurements (Fig. 7(b)) as a function of potential.

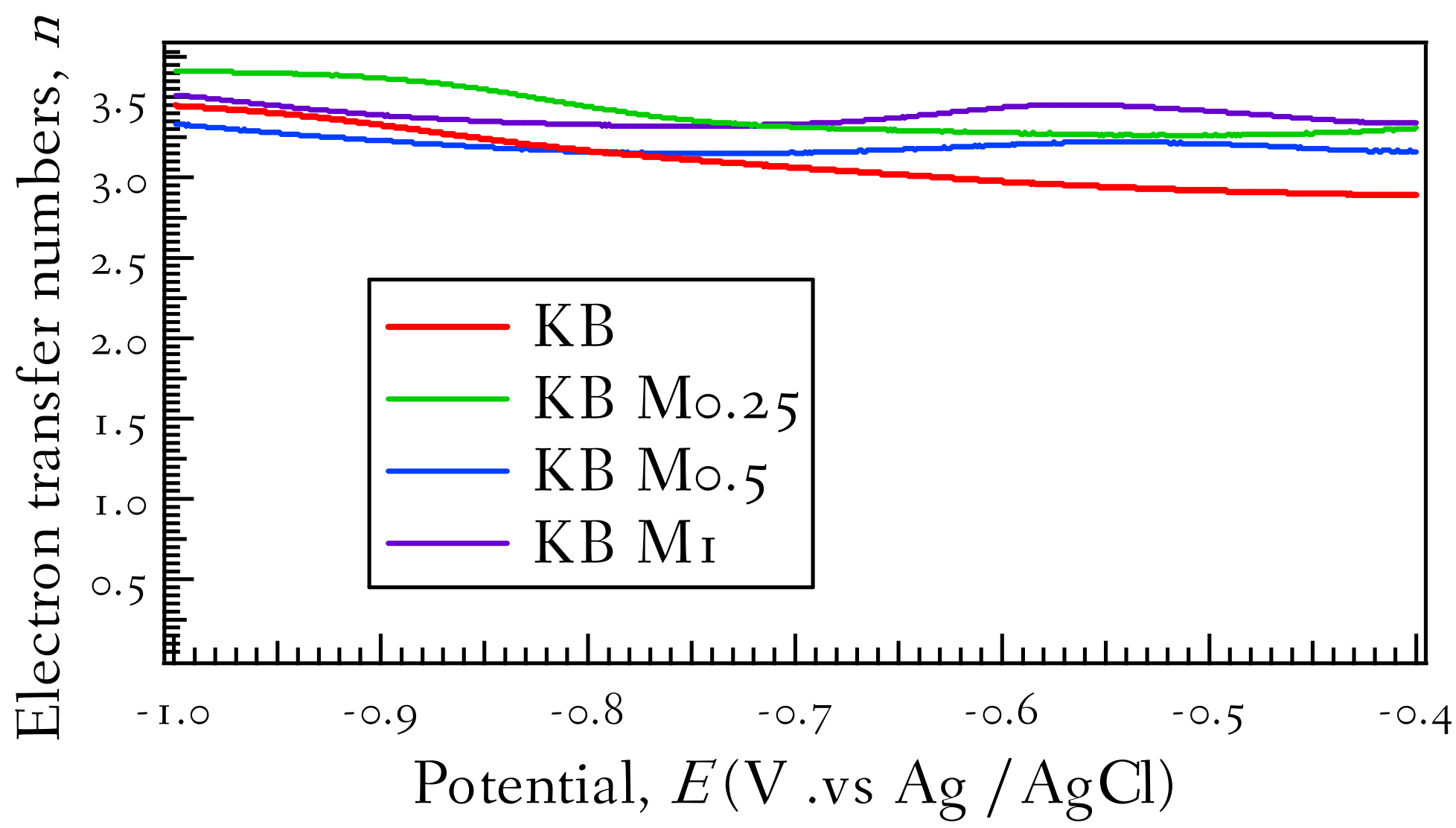

Figure S11: Change in hydrogen peroxide (H_2O_2) formation rate (%) derived from rotating ring-disk electrode (RRDE) measurements (Fig. 7(b)) as a function of potential.

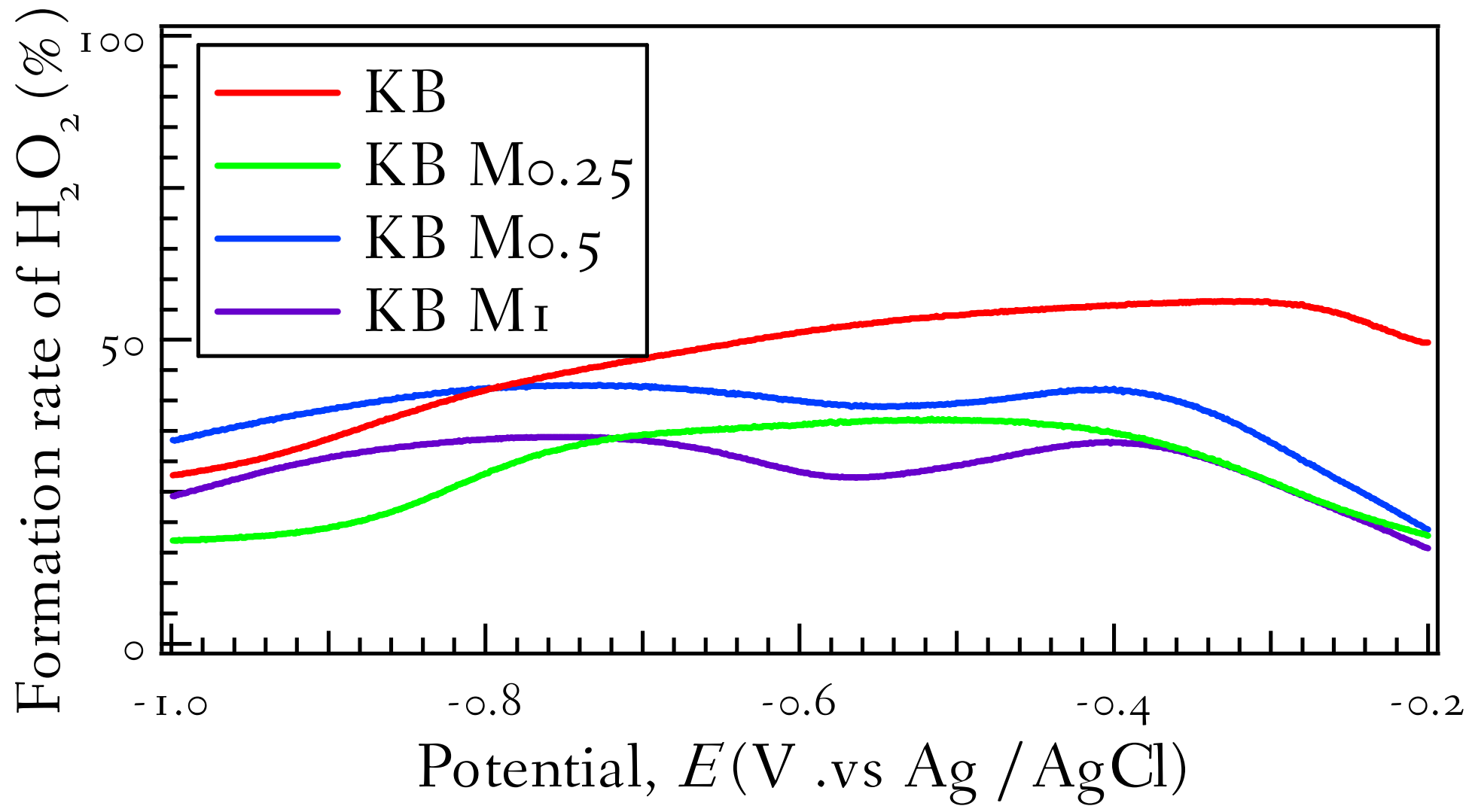

Figure S12: CV curves (in 0.1 M KOH) of the catalysts synthesized by SPP: (a) CNT-M0.25, (b) CNT-M0.5, and (c) CNT-M1.

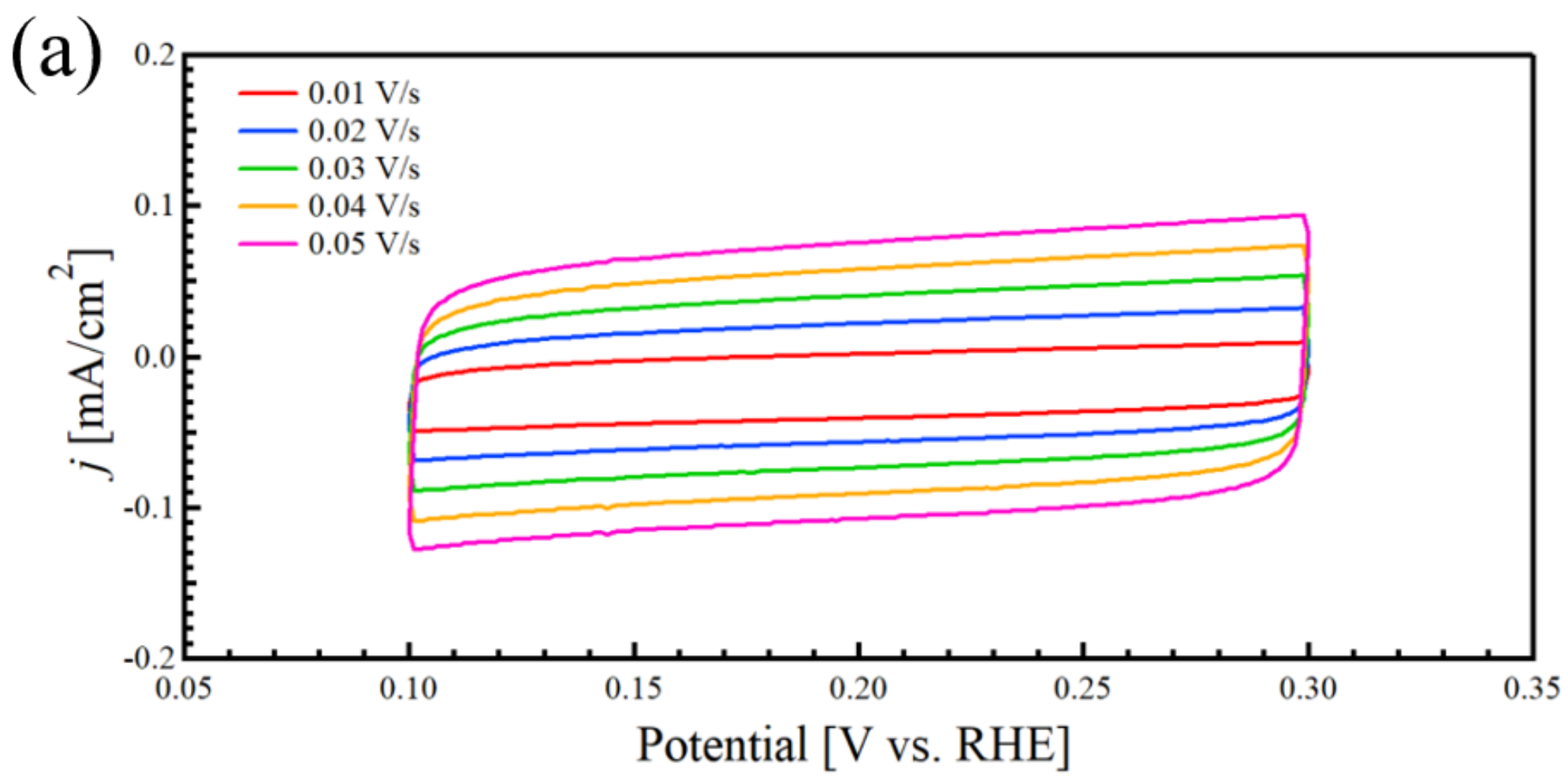

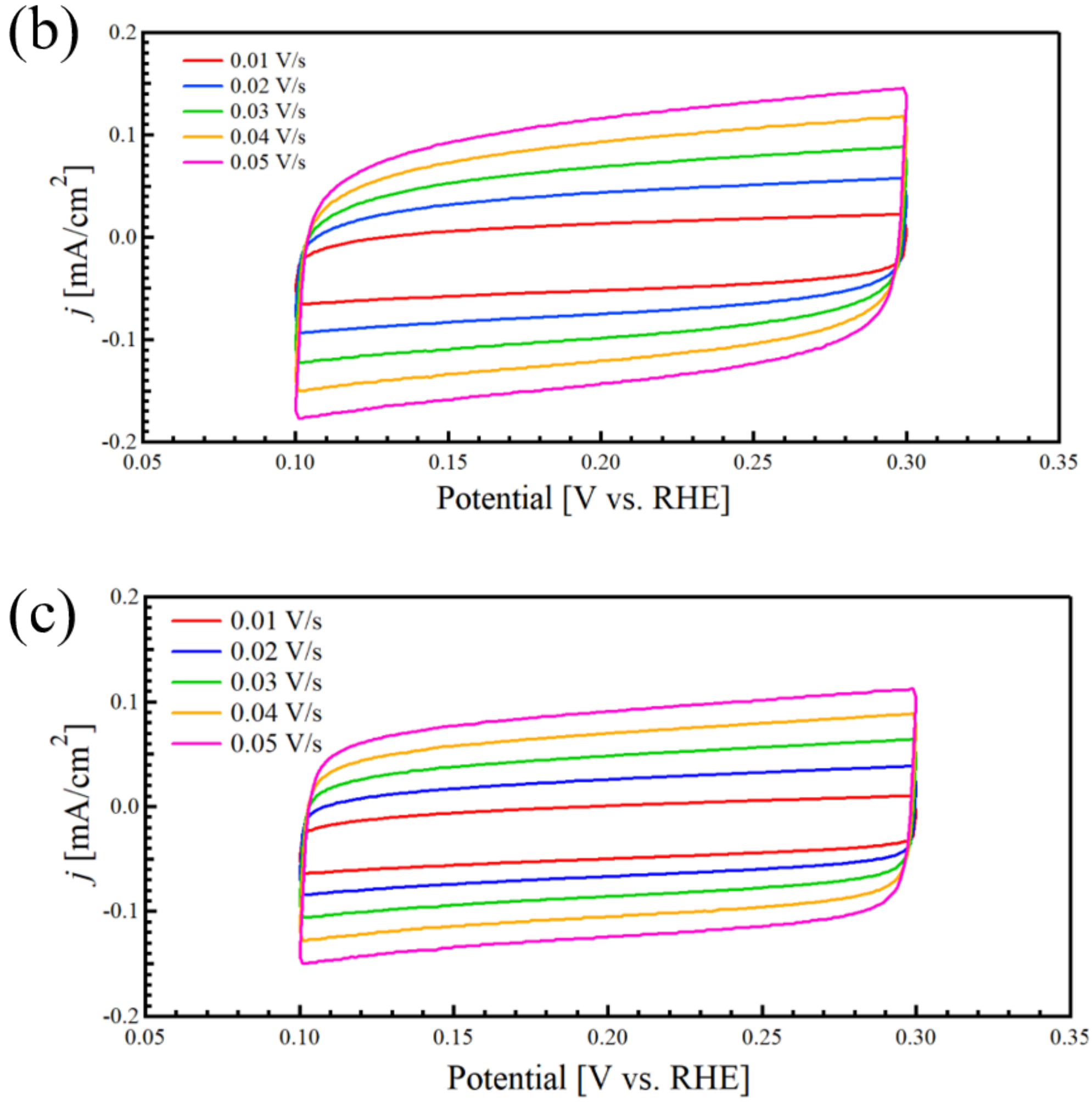

Figure S13: CV curves (in 0.1 M KOH) of the catalysts synthesized by SPP: (a) KB-M0.25, (b) KB-M0.5, and (c) KB-M1.

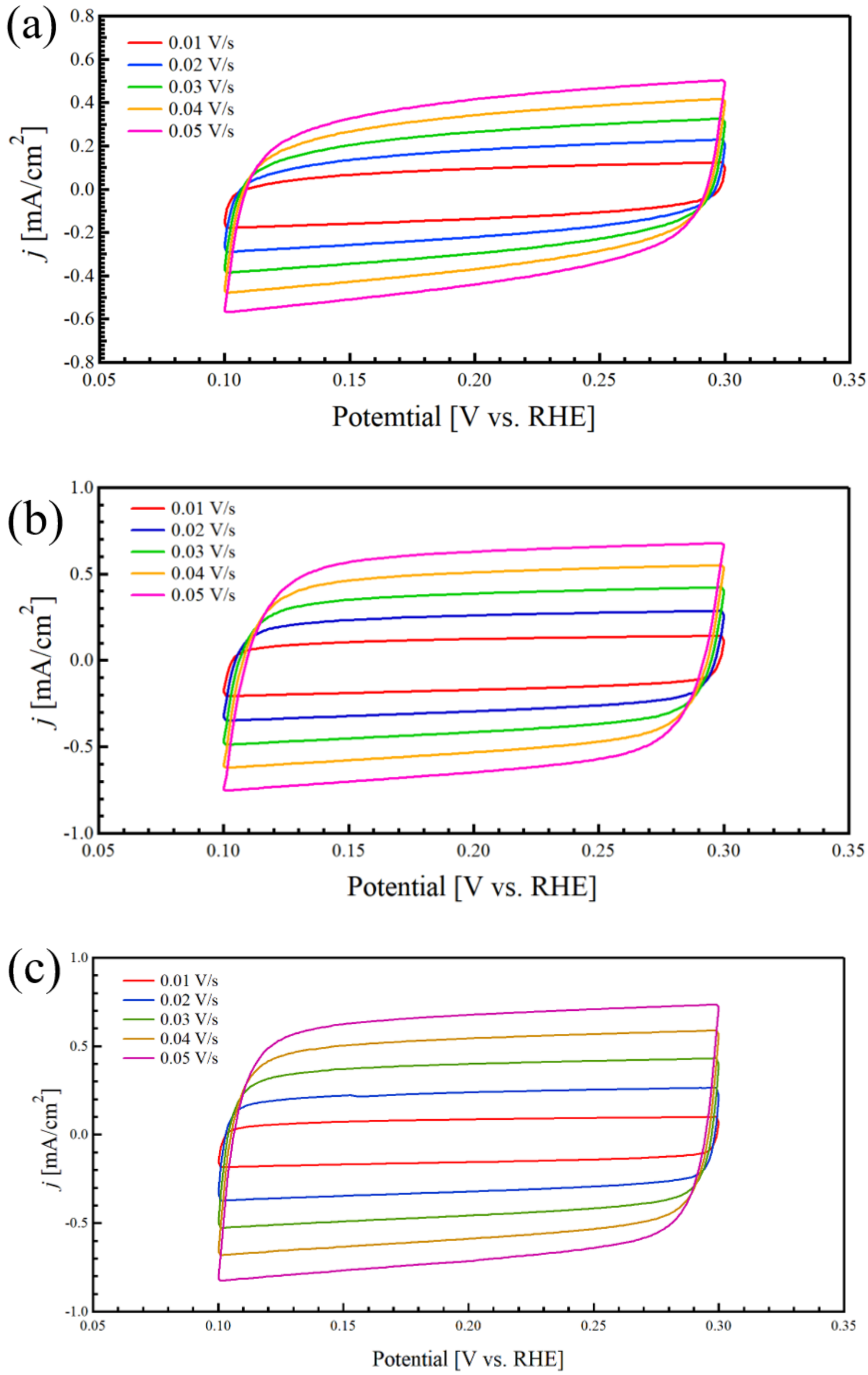

Figure S14: ORR LSV curves in 0.1 M KOH for CNT-M0.25, CNT-M0.5, and CNT-M1. The geometrically normalized current density was divided by the dimensionless capacitance-

derived apparent area ratio reported in Table S3. This empirical normalization is not interpreted as a measure of intrinsic Mn-site activity.

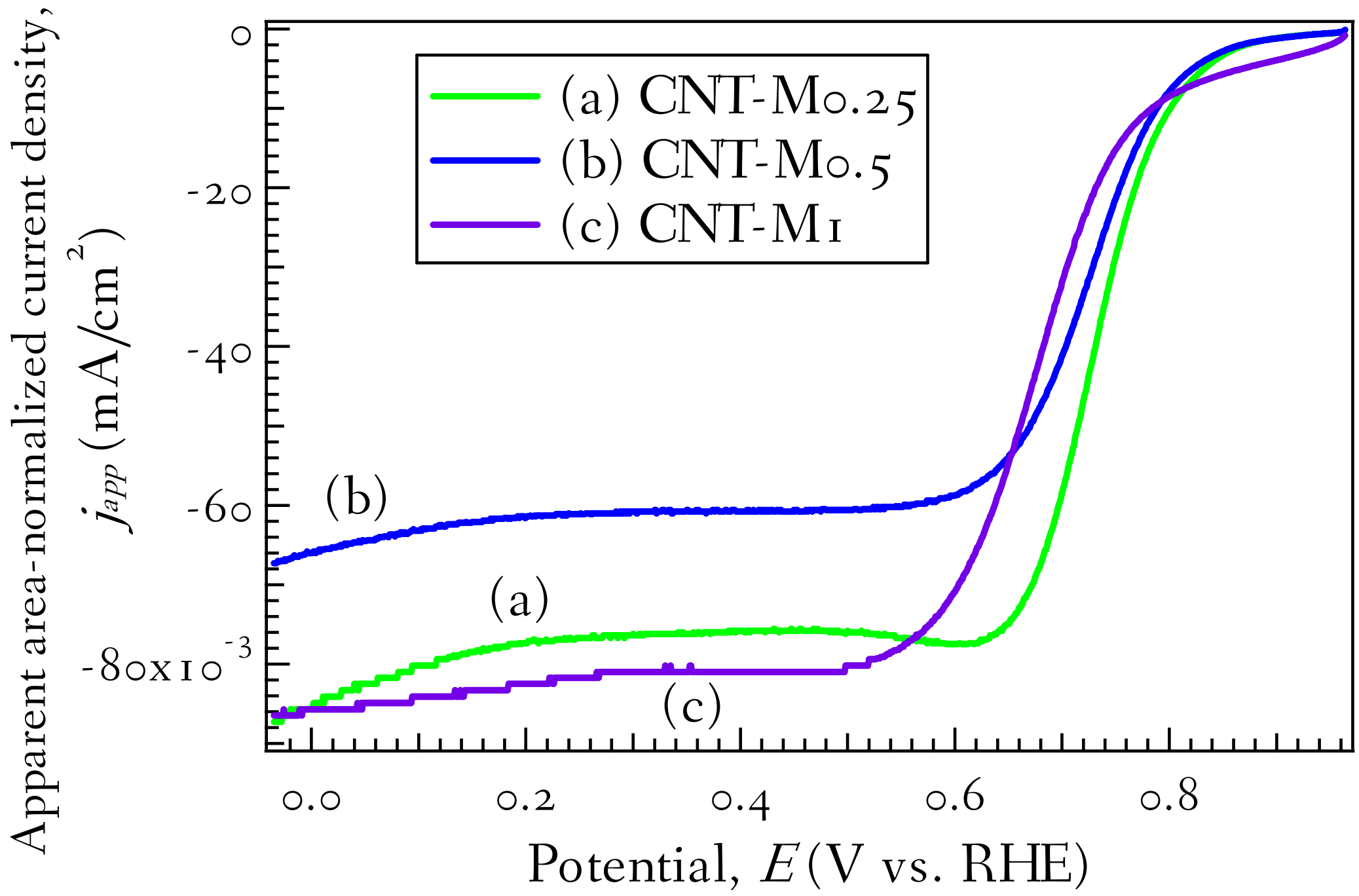

Figure S15: ORR LSV curves in 0.1 M KOH for KB-M0.25, KB-M0.5, and KB-M1. The geometrically normalized current density was divided by the dimensionless capacitance-

derived apparent area ratio reported in Table S3. This empirical normalization is not interpreted as a measure of intrinsic Mn-site activity.

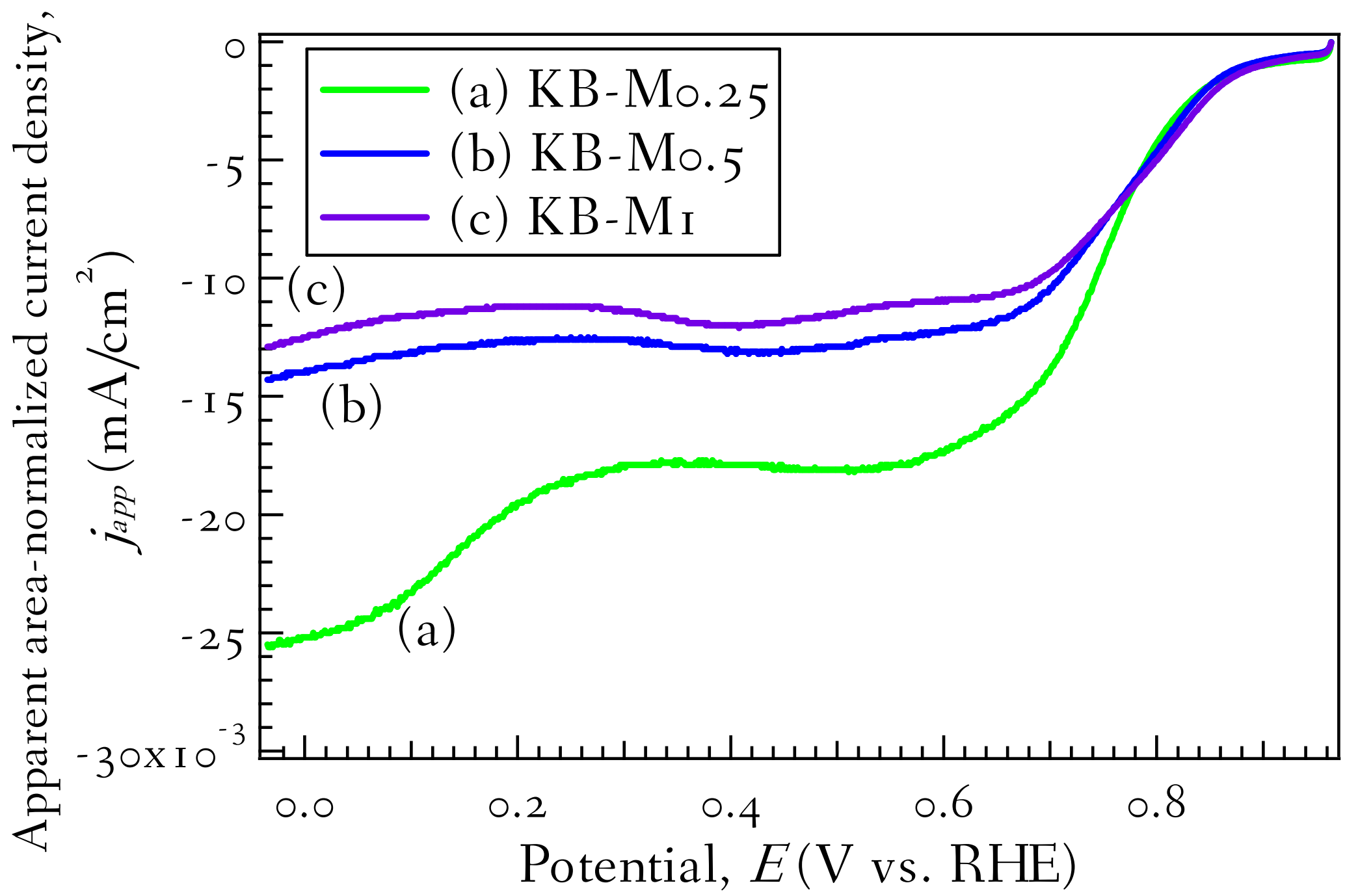

Figure S16: Raman spectra of (a) CNT-M0.5 and (b) KB-M1 before and after the 20,000 s CA test at 0.464 V vs. RHE in O_2-saturated 0.1 M KOH at 1500 rpm. Post-test samples were

recovered as described in Section 2.3.

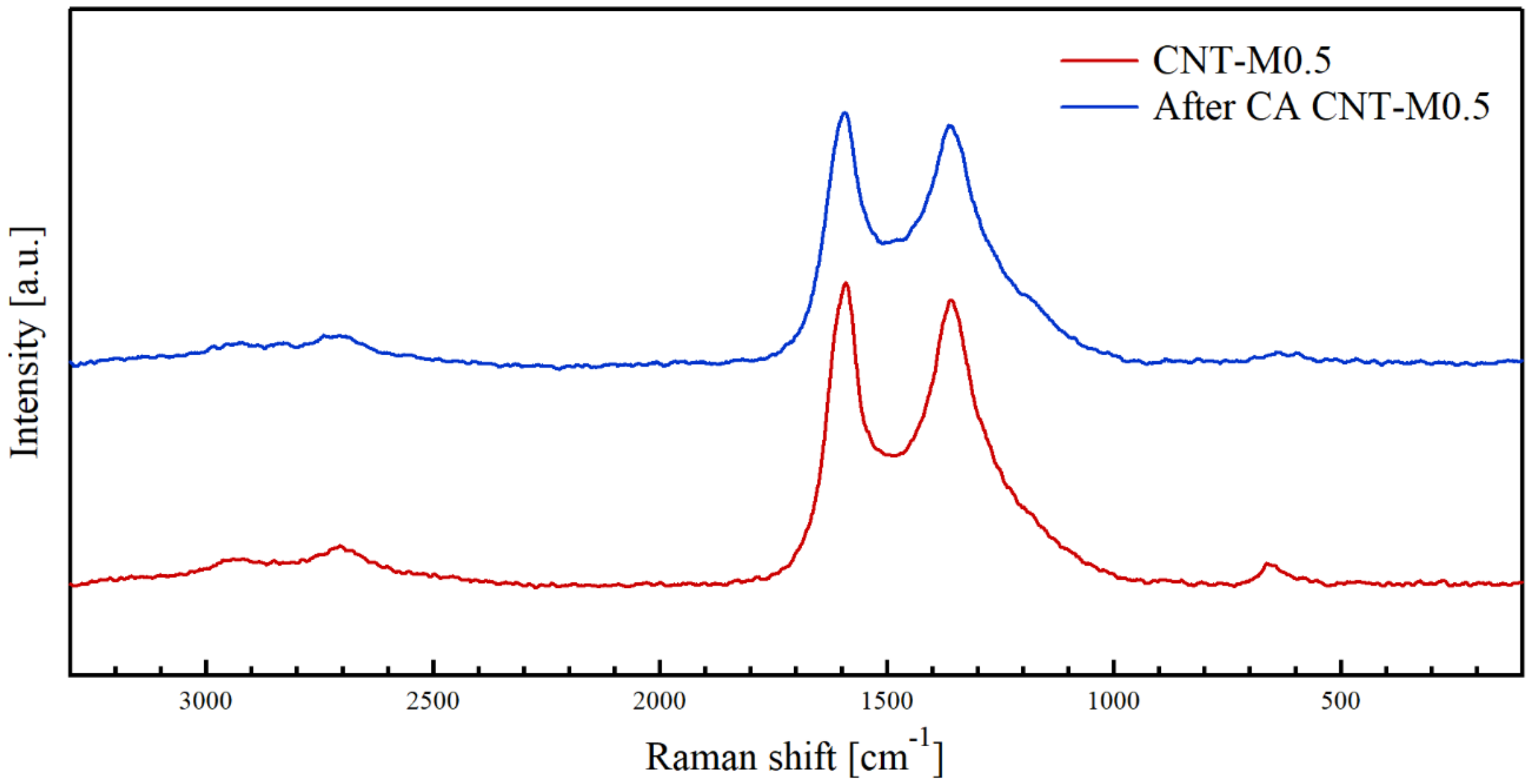

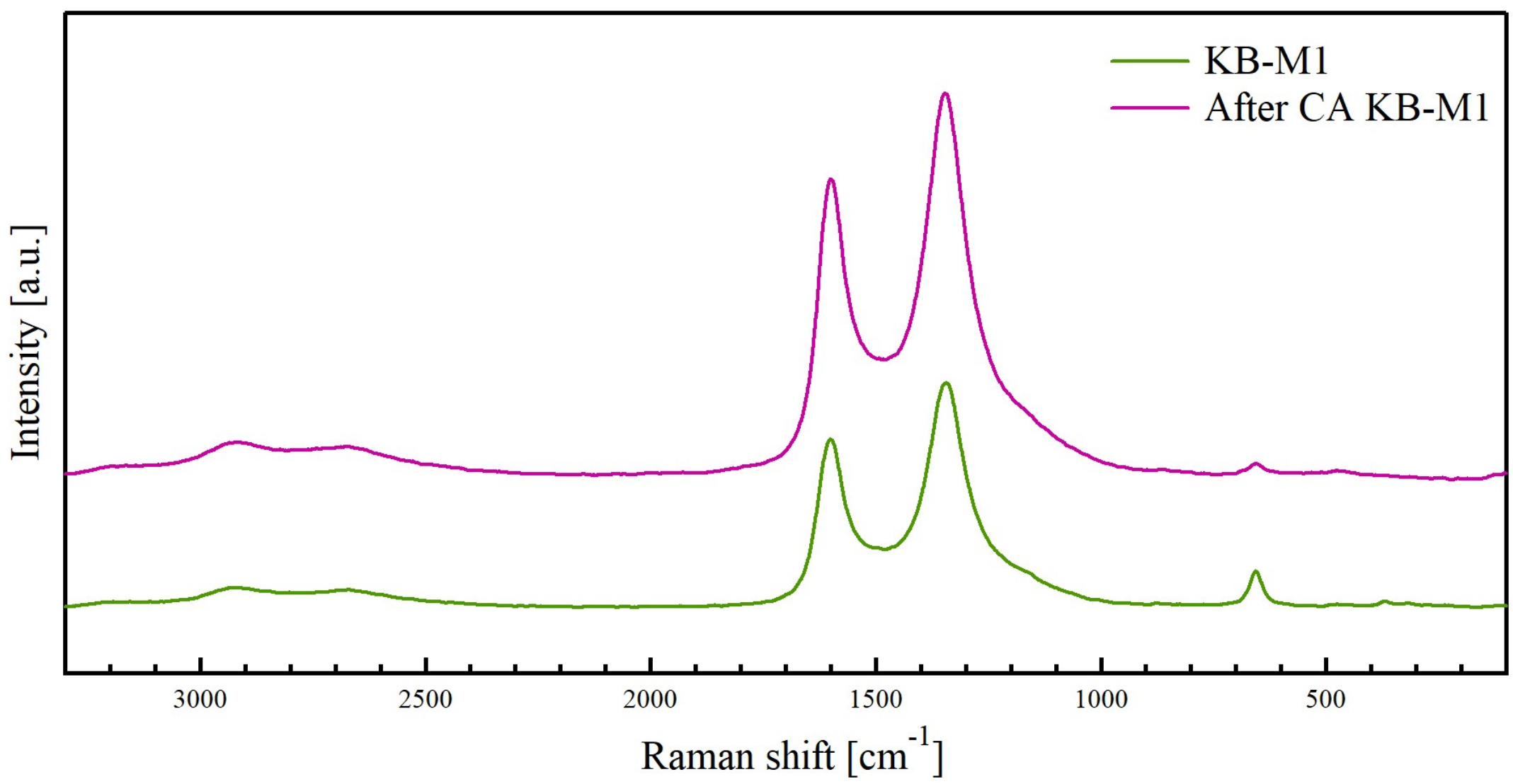

Figure S17: Post-CA SEM images of (a) CNT-M0.5 and (b) KB-M1 and corresponding Mn, O, and C EDS maps of (c) CNT-M0.5 and (d) KB-M1. After the 20,000 s CA test, the catalyst-

coated electrodes were rinsed in ultrapure water for 10 min while rotating at 1500 rpm, dried under ambient conditions, and the catalyst layers were detached for analysis.

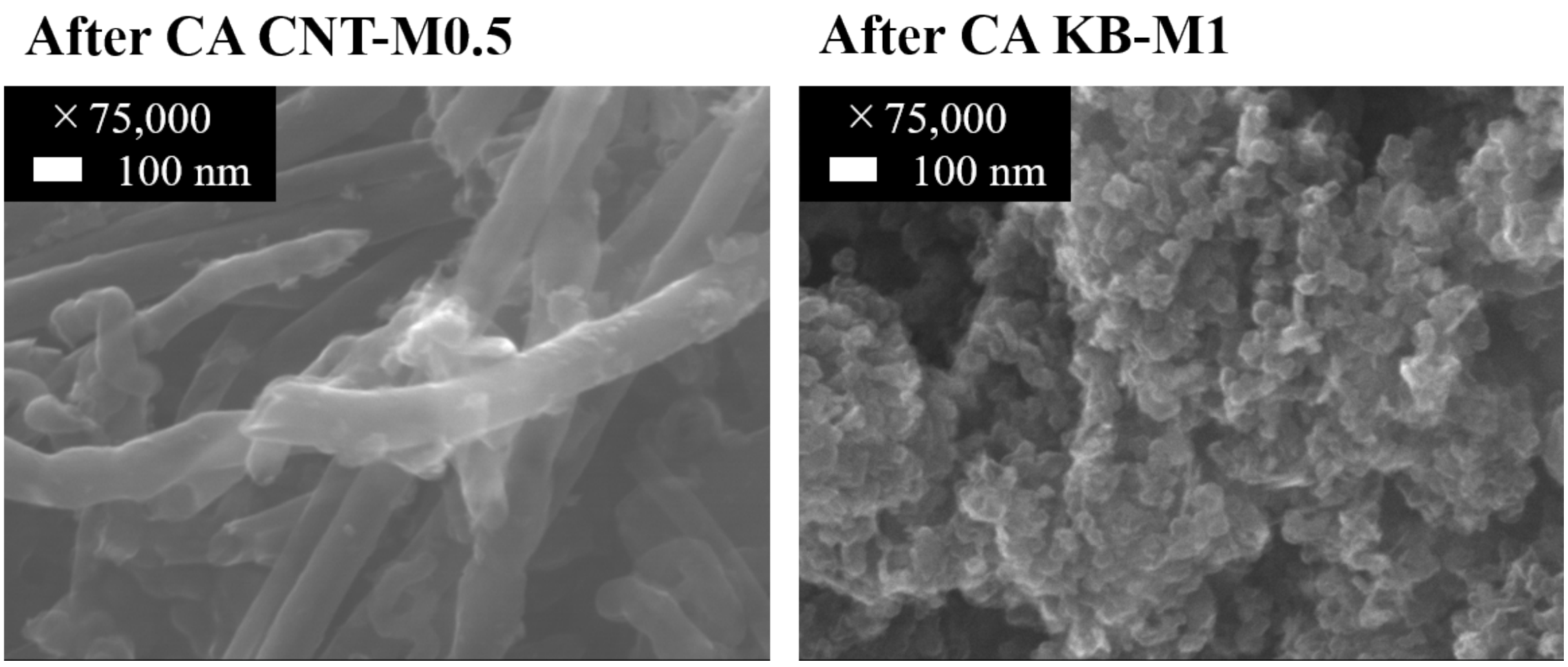

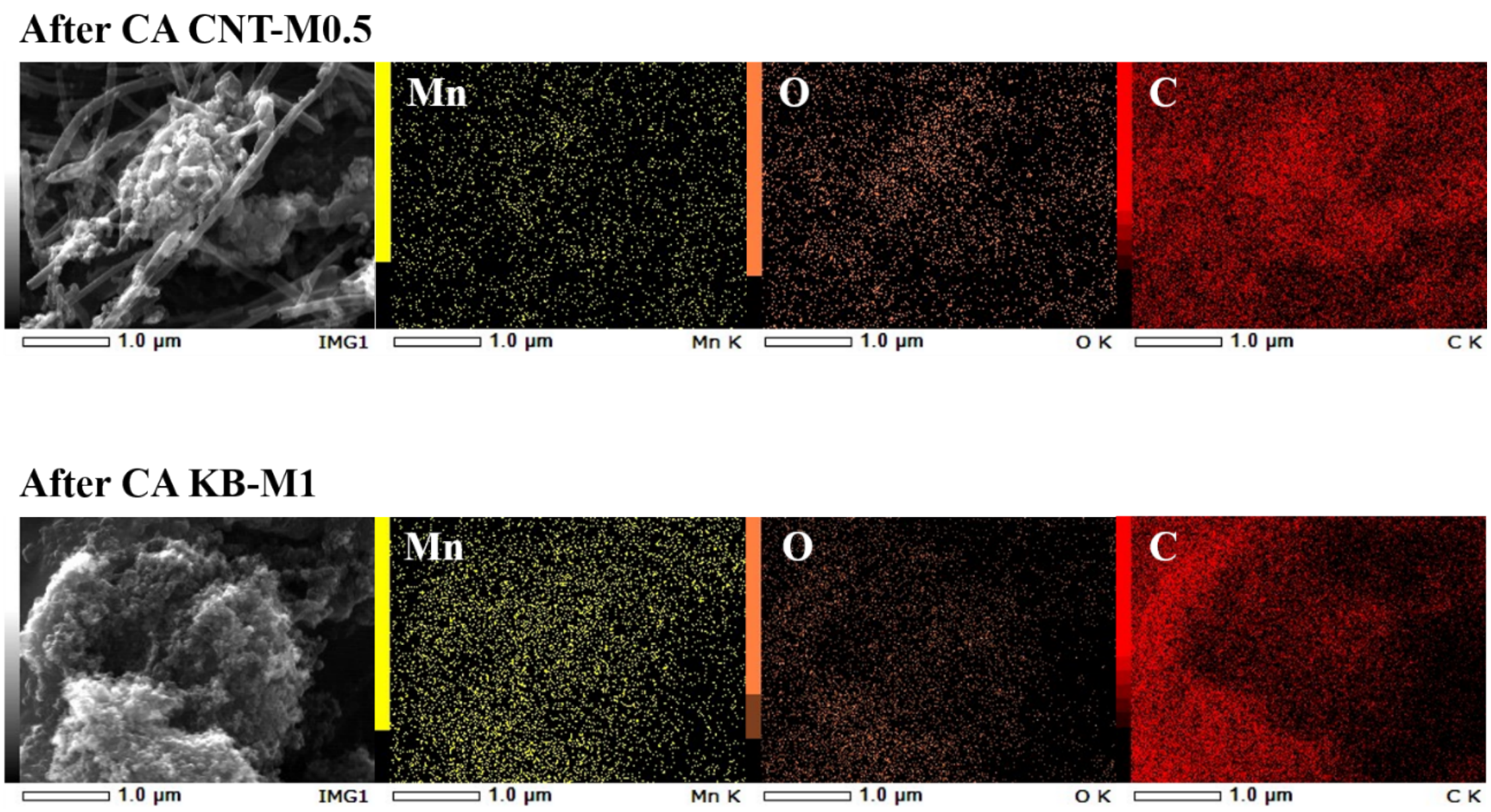

Supplement: Supplementary file 1 [file RA-016-D6RA06750F-s001.pdf]
